# Supplementary material for: Impaired IFNγ responsiveness of monocyte-derived lung cells limits immunity to Mycobacterium tuberculosis
Source: Nat Commun. 2026 Jun 30;17:8131. doi: 10.1038/s41467-026-75096-4 (PMC13458731; doi:10.1038/s41467-026-75096-4)
Supplement: Supplementary file 1 [file 41467_2026_75096_MOESM1_ESM.pdf]

Supplementary information

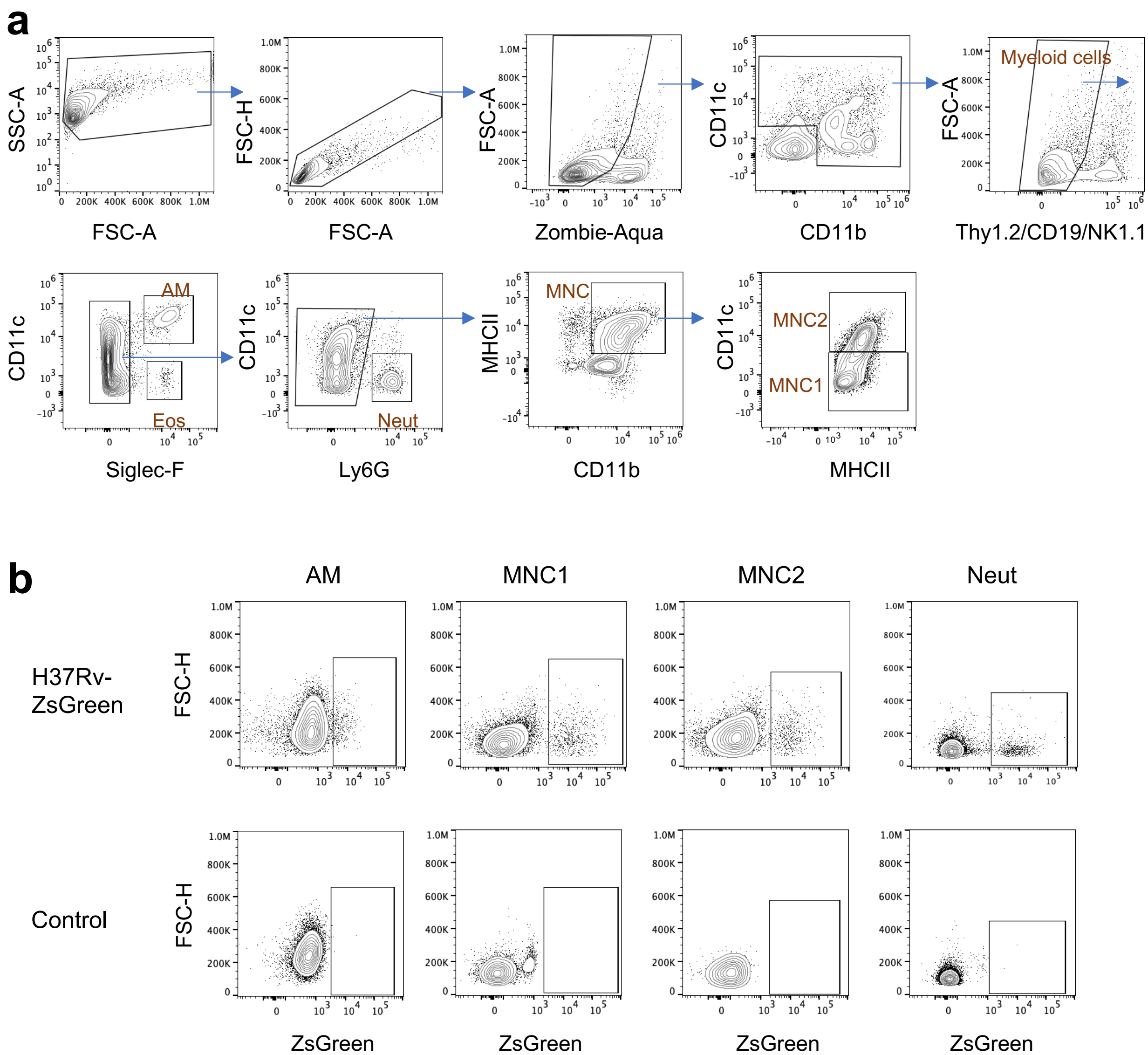

Supplementary Fig. 1: Flow gating strategy for lung cell populations.
(**a**) Flow cytometry plots for identifying alveolar macrophages (AM), monocyte-derived cells (MNC1, MNC2), and neutrophils (Neut) from Mtb-infected mouse lungs.
(**b**) Representative plots of infected cells for each phagocyte subset from mice infected with H37Rv-ZsGreen (28 dpi).

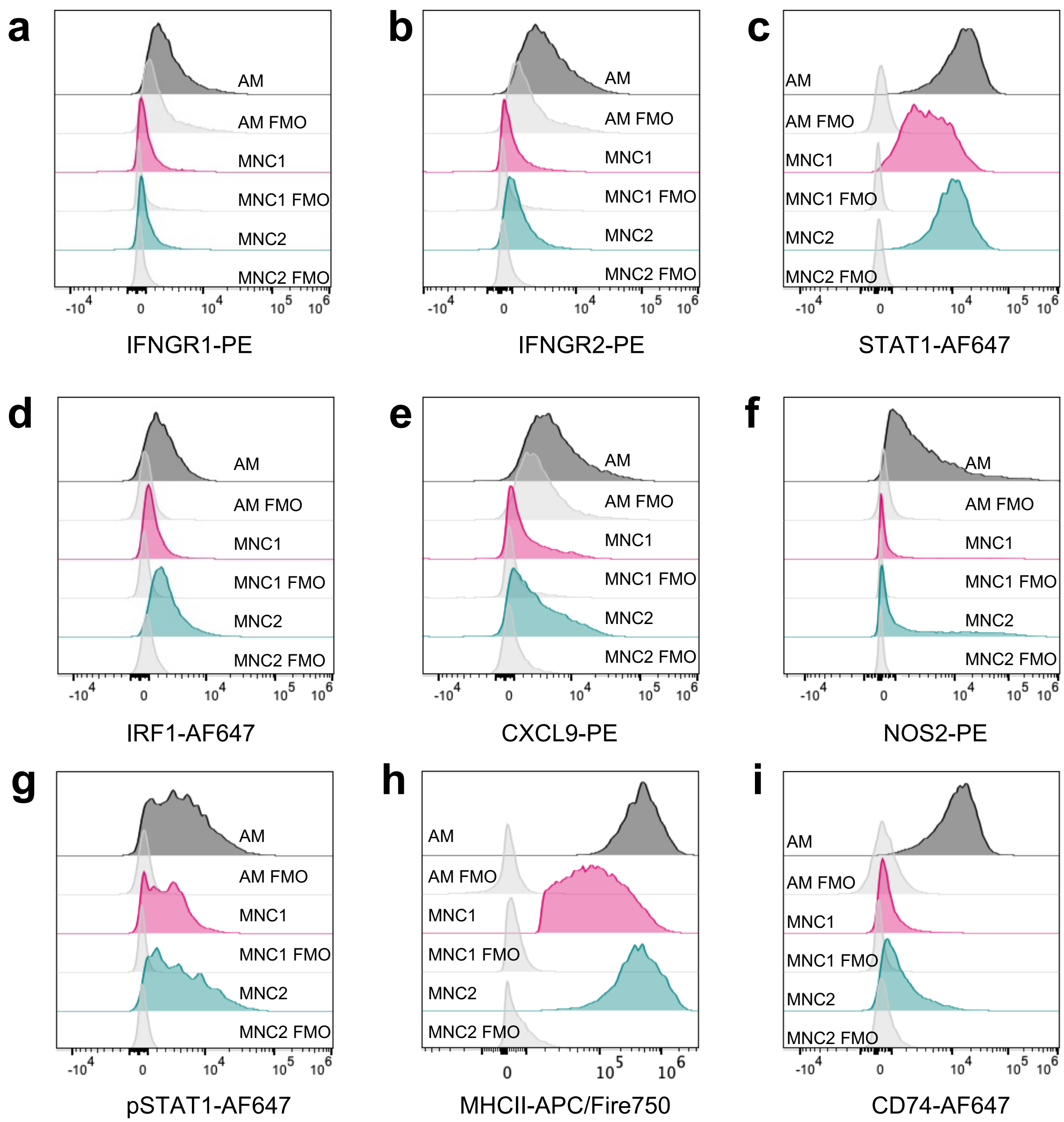

Supplementary Fig. 2: Histograms of proteins analyzed by flow cytometry for lung MNP subsets. FMO: fluorescence minus one.

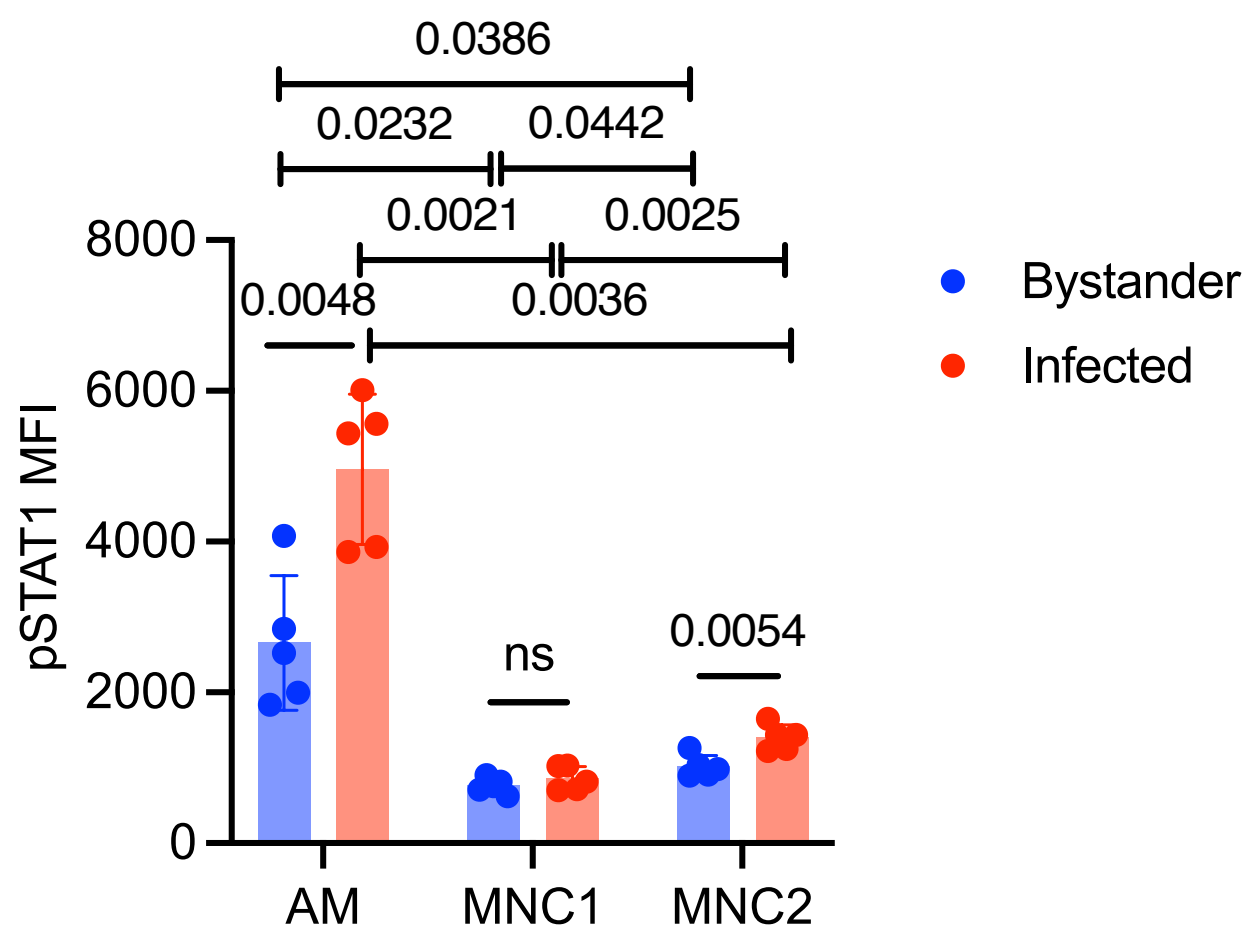

Supplementary Fig. 3: Impaired IFN-γ responsiveness of MNC1 cells compared to other subsets in bystander or infected cells.
C57BL/6 mice were infected with Mtb H37Rv-ZsGreen for 28 days (n = 5 mice). Lung single-cell suspensions were harvested and treated with 20 ng/mL IFN-γ for 15 min and subjected to pSTAT1 flow cytometry analysis. Results for bystander subsets (ZsGreen^{-}) and infected subsets (ZsGreen^{+}) are presented as mean ± SD. Representative data from 3 independent experiments are shown. Statistical significance was determined by Brown-Forsythe and Welch ANOVA tests with Dunnett's T3 multiple comparisons test (between subsets) or unpaired t-test (bystander versus infected subset); ns = not significant (p>0.05). Source data are provided as a Source Data file.

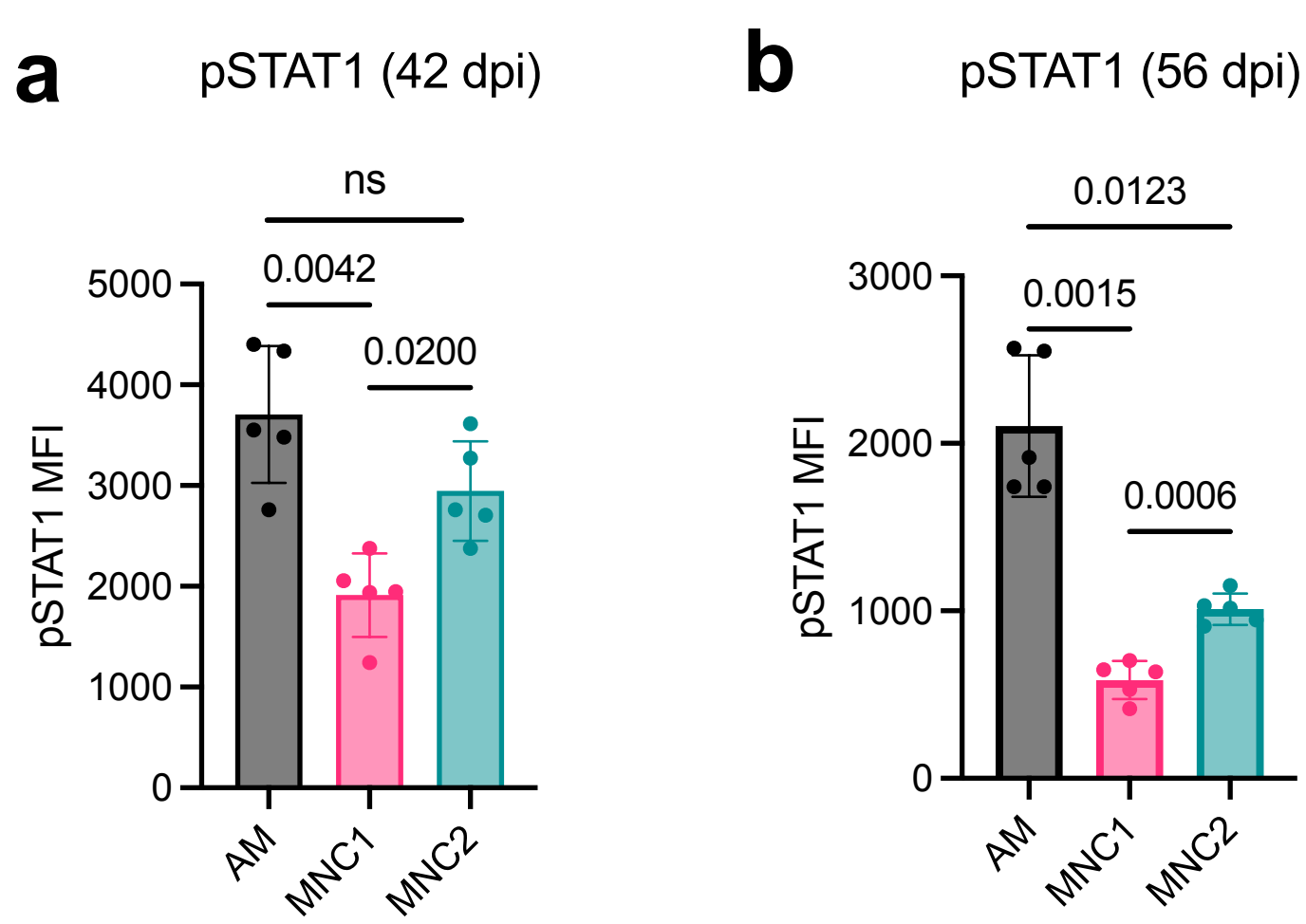

Supplementary Fig. 4: MNC1 cells are less responsive to IFN-γ at multiple time points of chronic Mtb infection.
C57BL/6 mice were infected with Mtb H37Rv-ZsGreen for 42 days (**a**) or 56 days (**b**) (n = 5 mice for both time points). Lung single-cell suspensions were incubated with 20 ng/mL IFN-γ for 15 min and subjected to pSTAT1 flow cytometry analysis. Results are presented as mean ± SD. Statistical significance was calculated by Brown-Forsythe and Welch ANOVA tests with Dunnett's T3 multiple comparisons test; ns = not significant (p>0.05). Source data are provided as a Source Data file.

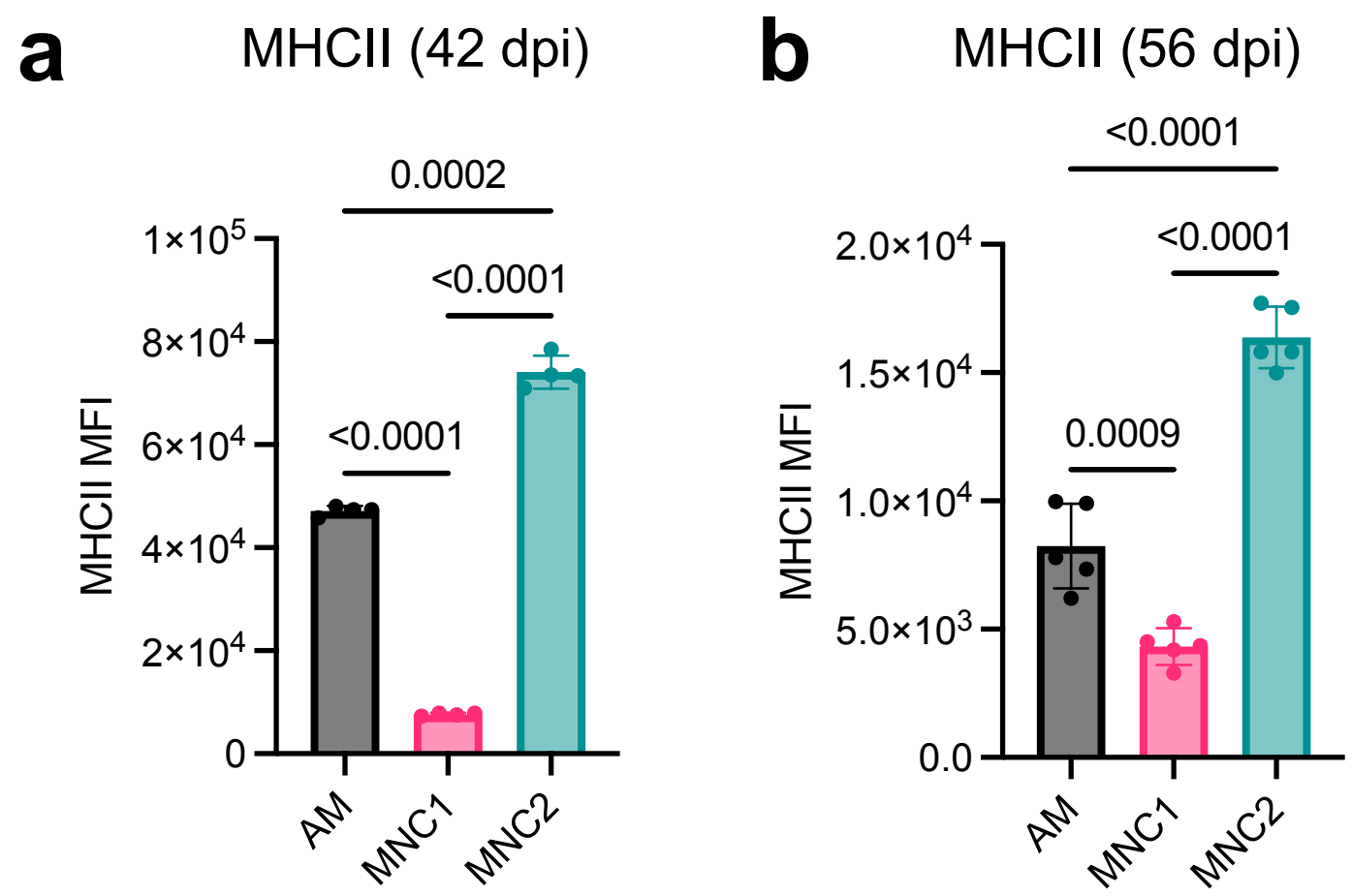

Supplementary Fig. 5: MHC-II expression level in MNP subsets at multiple time points of chronic Mtb infection. C57BL/6 mice were infected with Mtb H37Rv-ZsGreen for 42 days (**a**) or 56 days (**b**). Lung cells were harvested for flow cytometry analysis. Results are shown as mean ± SD. Representative data from 2 independent experiments are shown. Statistical significance was calculated by Brown-Forsythe and Welch ANOVA tests with Dunnett's T3 multiple comparisons test. Source data are provided as a Source Data file.

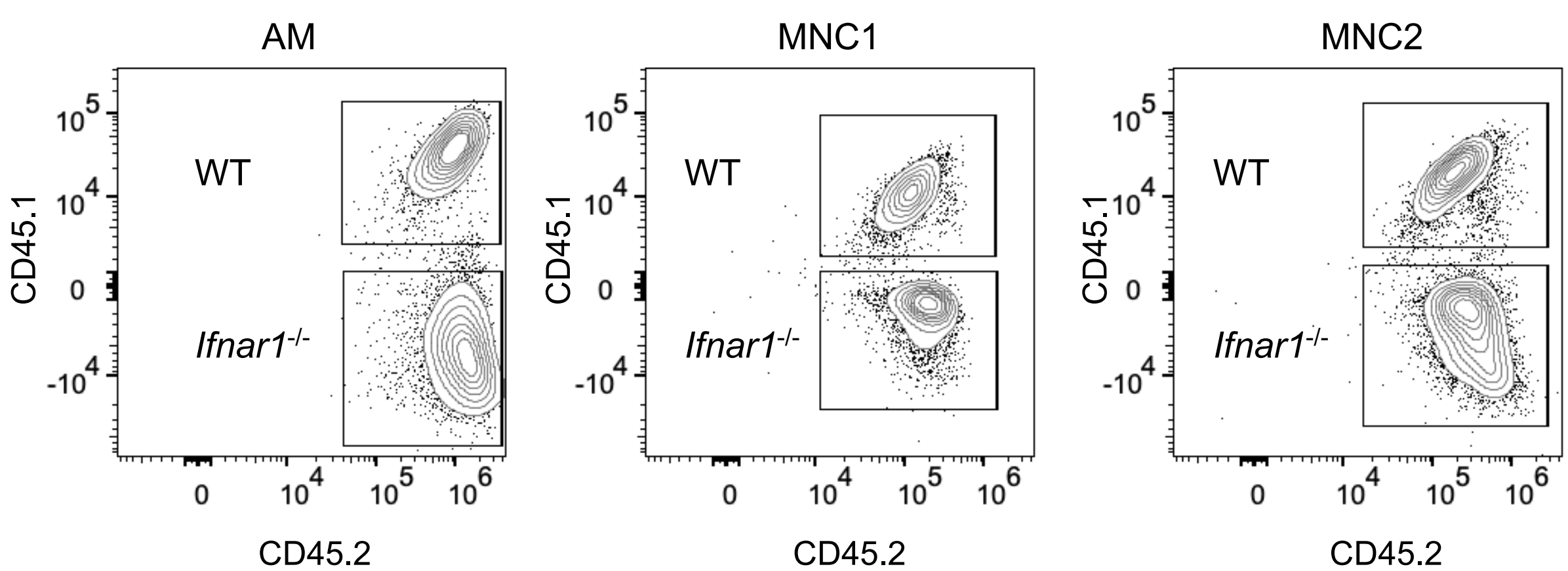

Supplementary Fig. 6: Gating strategy for WT and *Ifnar1*$^{-/-}$ subsets from mixed bone marrow chimeric mice. Gating of AM, MNC1, and MNC2 is shown in Figure S1.

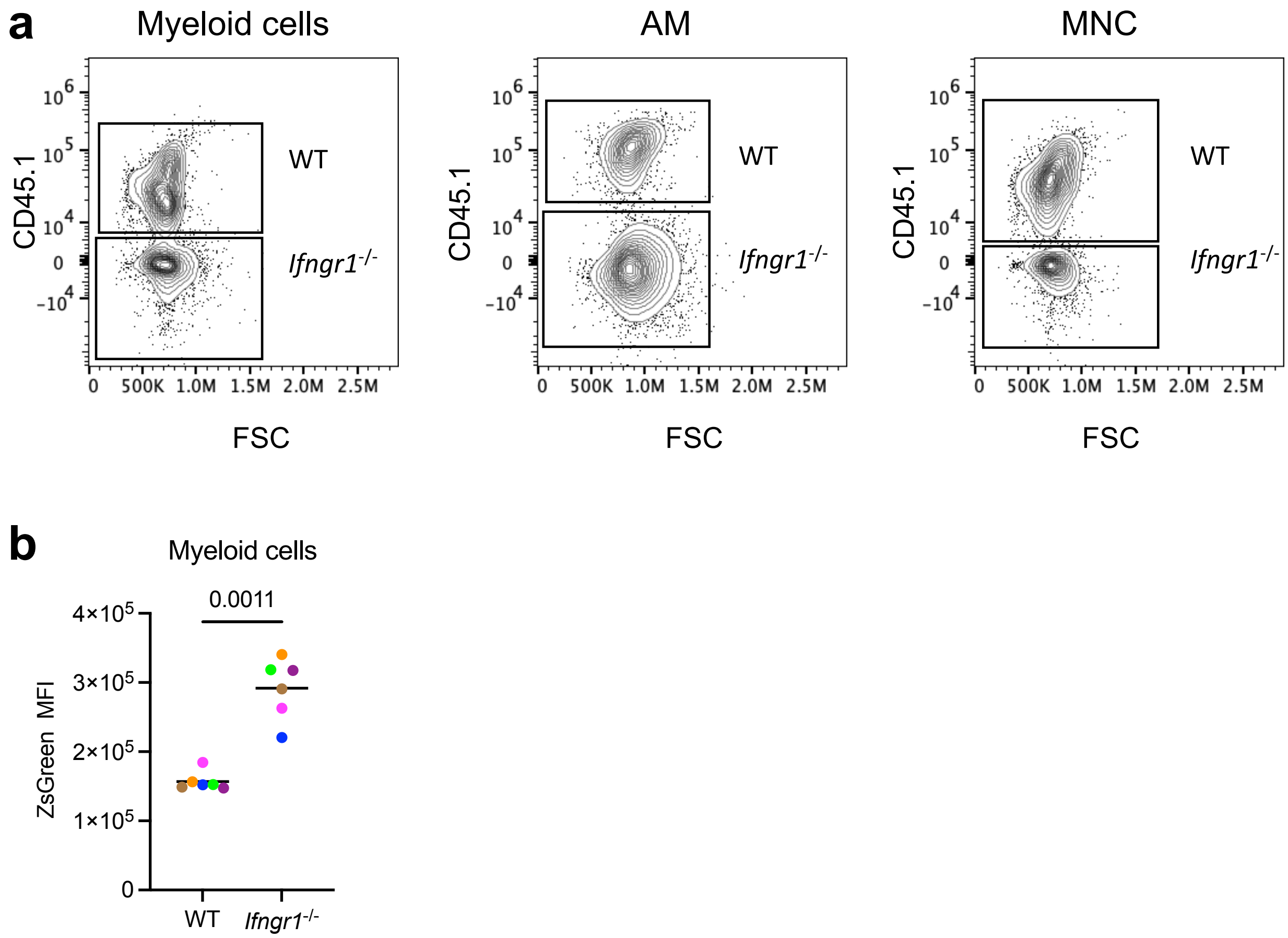

Supplementary Fig. 7: Gating strategy and Mtb burden for WT and *Ifngr1*$^{-/-}$ myeloid cells.

(**a**) Gating strategy for WT and *Ifngr1*$^{-/-}$ subsets. Gating of myeloid cells, AM, and MNC is shown in Figure S1.

(**b**) Mtb burden for WT and *Ifngr1*$^{-/-}$ myeloid cells (n = 5 mice). Each dot represents a single mouse. Each mouse is represented by a unique color. Statistical significance was calculated by two-tailed unpaired t-test. Source data are provided as a Source Data file.

Supplementary Table 1: IFN-γ responses differ in MNC1 and other MNP subsets. *p*-value was calculated by two-tailed Fisher's exact test, and the adjusted *p*-value was determined by Benjamini-Hochberg correction.

Category	# of DEG in category	# of genes in category	term	*p*-value	Adjusted *p*-value	MNC1 vs
GO:0034341	64	135	response to interferon-gamma	1.16E-05	4.36E-02	AM
GO:0034341	70	135	response to interferon-gamma	2.29E-13	1.09E-09	MMC2
GO:0071346	56	108	cellular response to interferon-gamma	9.33E-11	4.32E-07	MMC2

Supplementary Table 2: MHC-II Antigen presentation pathways differ in MNC1 and other MNP subsets. *p*-value was calculated by two-tailed Fisher's exact test, and the adjusted *p*-value was determined by Benjamini-Hochberg correction.

Category	# of DEG in category	# of genes in category	term	*p*-value	Adjusted *p*-value	MNC1 vs
GO:0002495	17	21	antigen processing and presentation of peptide antigen via MHC class II	5.63E-07	2.24E-03	AM
GO:0002495	16	21	antigen processing and presentation of peptide antigen via MHC class II	1.00E-07	4.35E-04	MMC2
GO:0002504	19	23	antigen processing and presentation of peptide or polysaccharide antigen via MHC class II	9.15E-08	3.74E-04	AM
GO:0002504	17	23	antigen processing and presentation of peptide or polysaccharide antigen via MHC class II	1.21E-07	5.25E-04	MMC2
GO:0019886	15	19	antigen processing and presentation of exogenous peptide antigen via MHC class II	4.85E-06	1.86E-02	AM
GO:0019886	14	19	antigen processing and presentation of exogenous peptide antigen via MHC class II	1.28E-06	5.34E-03	MMC2

Supplementary Table 3: Antibodies for flow cytometry

Antibodies	Source	Identifier
Brilliant Violet 421 anti-mouse Ly-6G Antibody	BioLegend	Cat#127627; RRID: AB_10897944
Brilliant Violet 605 anti-mouse CD11c Antibody	BioLegend	Cat#117334; RRID: AB_2562415
Brilliant Violet 711 anti-mouse/human CD11b Antibody	BioLegend	Cat#101242; RRID: AB_2563310
PE/Cyanine5 anti-mouse CD90.2 (Thy1.2) Antibody	BioLegend	Cat#105314; RRID: AB_313185
PE/Cyanine5 anti-mouse CD19 Antibody	BioLegend	Cat#115510; RRID: AB_313645
PE/Cyanine5 anti-mouse NK-1.1 Antibody	BioLegend	Cat#108716; RRID: AB_493590
PE/Cyanine7 anti-mouse Ly-6C Antibody	BioLegend	Cat#128018; RRID: AB_1732082
PE anti-mouse I-A/I-E Antibody	BioLegend	Cat#107608; RRID: AB_313323
Alexa Fluor® 647 anti-mouse I-A/I-E Antibody	BioLegend	Cat#107618; RRID: AB_493525
APC/Fire™ 750 anti-mouse I-A/I-E Antibody	BioLegend	Cat#107652; RRID: AB_2616729
PE Rat Anti-Mouse Siglec-F	BD Biosciences	Cat#552126; RRID: AB_394341
Alexa Fluor® 647 Rat Anti-Mouse Siglec-F	BD Biosciences	Cat#562680; RRID: AB_2687570
CXCL9 (MIG) Monoclonal Antibody (MIG-2F5.5), PE,	eBioscience	Cat# 12-3009-80; RRID: AB_891582
IRF-1 (D5E4) Rabbit mAb, Alexa Fluor 647	Cell Signaling Technology	Cat# 14105S; RRID: AB_2798393
Phospho-Stat1 (Tyr701) (58D6) Rabbit mAb, Alexa Fluor 647	Cell Signaling Technology	Cat# 8009S; RRID: AB_10860764
Alexa Fluor 647 Mouse anti-Total Stat1 (N-Terminus)	BD Biosciences	Cat#558560; RRID: AB_647143
Purified Rat Anti-Mouse CD16/CD32 (Mouse BD Fc Block™)	BD Biosciences	Cat#553142; RRID: AB_394656
PE anti-mouse IFNGR2 Antibody	Miltenyi Biotec	Cat# 130-105-671; RRID:AB_2652252
PE anti-mouse IFNGR2 Antibody	BioLegend	Cat# 113604; RRID: AB_313561
CD119 (IFN gamma Receptor 1) Monoclonal Antibody (2E2), PE	Invitrogen	Cat# 12-1191-82; RRID:AB_1210730
iNOS Monoclonal Antibody (CXNFT), PE	eBioscience	Cat# 12-5920-82; RRID: AB_2572642
