# Supplementary material for: Impaired IFNγ responsiveness of monocyte-derived lung cells limits immunity to Mycobacterium tuberculosis
Source: Nat Commun. 2026 Jun 30;17:8131. doi: 10.1038/s41467-026-75096-4 (PMC13458731; doi:10.1038/s41467-026-75096-4)
Supplement: Supplementary file 2 [file 41467_2026_75096_MOESM2_ESM.pdf]

nature portfolio

Corresponding author(s): Weihao Zheng, Joel D. Ernst

Last updated by author(s): Jun 7, 2026

Reporting Summary

Nature Portfolio wishes to improve the reproducibility of the work that we publish. This form provides structure for consistency and transparency in reporting. For further information on Nature Portfolio policies, see our Editorial Policies and the Editorial Policy Checklist.

Statistics

For all statistical analyses, confirm that the following items are present in the figure legend, table legend, main text, or Methods section.

n/a	Confirmed	
[ ]	[x]	The exact sample size (*n*) for each experimental group/condition, given as a discrete number and unit of measurement
[ ]	[x]	A statement on whether measurements were taken from distinct samples or whether the same sample was measured repeatedly
[ ]	[x]	The statistical test(s) used AND whether they are one- or two-sided<br>*Only common tests should be described solely by name; describe more complex techniques in the Methods section.*
[x]	[ ]	A description of all covariates tested
[ ]	[x]	A description of any assumptions or corrections, such as tests of normality and adjustment for multiple comparisons
[ ]	[x]	A full description of the statistical parameters including central tendency (e.g. means) or other basic estimates (e.g. regression coefficient) AND variation (e.g. standard deviation) or associated estimates of uncertainty (e.g. confidence intervals)
[ ]	[x]	For null hypothesis testing, the test statistic (e.g. *F*, *t*, *r*) with confidence intervals, effect sizes, degrees of freedom and *P* value noted<br>*Give P values as exact values whenever suitable.*
[x]	[ ]	For Bayesian analysis, information on the choice of priors and Markov chain Monte Carlo settings
[x]	[ ]	For hierarchical and complex designs, identification of the appropriate level for tests and full reporting of outcomes
[x]	[ ]	Estimates of effect sizes (e.g. Cohen's *d*, Pearson's *r*), indicating how they were calculated

Our web collection on statistics for biologists contains articles on many of the points above.

Software and code

Policy information about availability of computer code

Data collection	BD FACSDiva, Spectroflo version 3.1 and Sony MA900 Cell Sorter Software v3.1.0 were used to collect flow cytometry data.<br>Sony MA900 Cell Sorter Software v3.1.0 was used to sort cells for experiments in this study.<br>Immunofluorescence imaging was performed using a Leica DMRB fluorescence upright microscope and SPOT basic software.
Data analysis	Quantification of MFI from immunofluorescence images was done using Fiji-ImageJ (2.16.0). Flow cytometry data were analyzed using FlowJo (version 10.8.1). GraphPad Prism software (version 10) was used for graphical presentation and statistical analysis.<br>RNA sequence processing and alignment: FastQC2 (v0.11.9) was used to generate quality-control reports of individual FASTQ files. Read were then aligned to the Mus musculus (house mouse) genome assembly GRCm38 (mm10) with the splice-aware STAR aligner using 'GeneCounts' quant mode with the Ensembl gene transfer format file (indexes were built using '—sjdbOverhang 50' and the gtf file). Biotypes were investigated using PICARD tools (v2.22.3).<br>Differential gene expression and pathway analysis: Differential gene expression analyses were done in R using the DESeq2 package (v1.26.0) which uses a negative binomial generalized linear model. Blinded variance stabilizing transformation was applied and t-sne plots were then generated with Rtsne (v0.15) using PCA and a perplexity of three. Differentially expressed genes were identified using a Benjamini-Hochberg corrected alpha of 0.05 and an absolute effect size of one. Kyoto Encyclopedia of Genes and Genomes (KEGG; v4.0) pathway analyses were done using the R package clusterProfiler (v3.14.3). Analyses of custom pathways considering the gene background and not were done using camera and roast (using 99990 rotations) from the limma package (v3.42.2) respectively.

For manuscripts utilizing custom algorithms or software that are central to the research but not yet described in published literature, software must be made available to editors and reviewers. We strongly encourage code deposition in a community repository (e.g. GitHub). See the Nature Portfolio guidelines for submitting code & software for further information.

Data

Policy information about availability of data

All manuscripts must include a data availability statement. This statement should provide the following information, where applicable:

- Accession codes, unique identifiers, or web links for publicly available datasets
- A description of any restrictions on data availability
- For clinical datasets or third party data, please ensure that the statement adheres to our policy

All data are available in the main text, Supplementary Information file, and Source Data file. The RNA-seq data supporting the findings of this study are available in the Gene Expression Omnibus (GEO) under the accession number: GSE220147. Requests for resources, reagents, or further information should be directed to the corresponding authors. Source data are provided with this paper.

Research involving human participants, their data, or biological material

Policy information about studies with human participants or human data. See also policy information about sex, gender (identity/presentation), and sexual orientation and race, ethnicity and racism.

Reporting on sex and gender	n/a
Reporting on race, ethnicity, or other socially relevant groupings	n/a
Population characteristics	n/a
Recruitment	n/a
Ethics oversight	n/a

Note that full information on the approval of the study protocol must also be provided in the manuscript.

Field-specific reporting

Please select the one below that is the best fit for your research. If you are not sure, read the appropriate sections before making your selection.

[x] Life sciences [ ] Behavioural & social sciences [ ] Ecological, evolutionary & environmental sciences

For a reference copy of the document with all sections, see nature.com/documents/nr-reporting-summary-flat.pdf

Life sciences study design

All studies must disclose on these points even when the disclosure is negative.

Sample size	The sample size was determined based on established field standards and historical data from similar studies and pilot experiments. The sample size is sufficient to ensure robust statistical analysis and detect meaningful differences between groups.
Data exclusions	No data were excluded.
Replication	All experiments were performed at least in triplicate. All attempts were successful, and the results are consistent.
Randomization	Mice were randomly assigned to different groups.
Blinding	Blinding was not possible as all experimental groups were compared to the control group. To minimize investigator bias, all data analysis was performed using objective, standardized criteria.

Reporting for specific materials, systems and methods

We require information from authors about some types of materials, experimental systems and methods used in many studies. Here, indicate whether each material, system or method listed is relevant to your study. If you are not sure if a list item applies to your research, read the appropriate section before selecting a response.

Materials & experimental systems

n/a	Involved in the study
	[x] Antibodies
[x]	Eukaryotic cell lines
[x]	Palaeontology and archaeology
	[x] Animals and other organisms
[x]	Clinical data
[x]	Dual use research of concern
[x]	Plants

Methods

n/a	Involved in the study
[x]	ChIP-seq
	[x] Flow cytometry
[x]	MRI-based neuroimaging

Antibodies

Antibodies used

Brilliant Violet 421 anti-mouse Ly-6G Antibody BioLegend Cat#127627; RRID: AB_10897944
Brilliant Violet 605 anti-mouse CD11c Antibody BioLegend Cat#117334; RRID: AB_2562415
Brilliant Violet 711 anti-mouse/human CD11b Antibody BioLegend Cat#101242; RRID: AB_2563310
PE/Cyanine5 anti-mouse CD90.2 (Thy1.2) Antibody BioLegend Cat#105314; RRID: AB_313185
PE/Cyanine5 anti-mouse CD19 Antibody BioLegend Cat#115510; RRID: AB_313645
PE/Cyanine5 anti-mouse NK-1.1 Antibody BioLegend Cat#108716; RRID: AB_493590
PE/Cyanine7 anti-mouse Ly-6C Antibody BioLegend Cat#128018; RRID: AB_1732082
PE anti-mouse I-A/I-E Antibody BioLegend Cat#107608; RRID: AB_313323
Alexa Fluor® 647 anti-mouse I-A/I-E Antibody BioLegend Cat#107618; RRID: AB_493525
APC/Fire™ 750 anti-mouse I-A/I-E Antibody BioLegend Cat#107652; RRID: AB_2616729
PE Rat Anti-Mouse Siglec-F BD Biosciences Cat#552126; RRID:
AB_394341
Alexa Fluor® 647 Rat Anti-Mouse Siglec-F BD Biosciences Cat#562680; RRID:
AB_2687570
CXCL9 (MIG) Monoclonal Antibody (MIG-2F5.5), PE, eBioscience Cat# 12-3009-80; RRID: AB_891582
IRF-1 (D5E4) Rabbit mAb, Alexa Fluor 647 Cell Signaling Technology Cat# 14105S; RRID: AB_2798393
Phospho-Stat1 (Tyr701) (58D6) Rabbit mAb, Alexa Fluor 647 Cell Signaling Technology Cat# 8009S; RRID: AB_10860764
Alexa Fluor 647 Mouse anti-Total Stat1 (N-Terminus) BD Biosciences Cat#558560; RRID:
AB_647143
Purified Rat Anti-Mouse CD16/CD32 (Mouse BD Fc Block™) BD Biosciences Cat#553142; RRID:
AB_394656
PE anti-mouse IFNGR2 Antibody Miltenyi Biotec Cat# 130-105-671; RRID:AB_2652252
PE anti-mouse IFNGR2 Antibody BioLegend Cat# 113604; RRID:
AB_313561
CD119 (IFN gamma Receptor 1) Monoclonal Antibody (2E2), PE Invitrogen Cat# 12-1191-82; RRID:AB_1210730
iNOS Monoclonal Antibody (CXNFT), PE eBioscience Cat# 12-5920-82; RRID: AB_2572642

Validation

Antibodies used in this study are commercially available and are validated by the manufacturers.

Animals and other research organisms

Policy information about studies involving animals; ARRIVE guidelines recommended for reporting animal research, and Sex and Gender in Research

Laboratory animals

All animal experiments were approved by the Institutional Animal Care and Use Committee of the University of California, San Francisco (Protocol Number: AN206266), and were performed in accordance with the Guide for the Care and Use of Laboratory Animals of the National Institutes of Health and the Office of Laboratory Animal Welfare. 8-12-week-old C57BL/6 (Strain #:000664), Ifnar1-/- (Strain #:028288) and Ifngr1-/- (Strain #:003288) mice were obtained from Jackson Laboratory. Uninfected mice were housed under SPF condition. Infected mice were housed in ABSL3. Mice were housed under standard conditions, including a 12-hour dark/light cycle, ambient temperature of 20–24°C, and relative humidity of 45–65%, with ad libitum access to food and water.

Wild animals

The study did not involve wild animals.

Reporting on sex

Both sexes were used in this study. Extensive experiments and projects in our lab demonstrate no sex-specific differences in our murine TB models. Therefore, sex was not a consideration in this study; sample sex information has not been collected.

Field-collected samples

n/a

Ethics oversight

All animal experiments were approved by the Institutional Animal Care and Use Committee of University of California, San Francisco.

Note that full information on the approval of the study protocol must also be provided in the manuscript.

Plants

Seed stocks

n/a

Novel plant genotypes

n/a

Authentication

n/a

Flow Cytometry

Plots

Confirm that:

- [x] The axis labels state the marker and fluorochrome used (e.g. CD4-FITC).
- [x] The axis scales are clearly visible. Include numbers along axes only for bottom left plot of group (a 'group' is an analysis of identical markers).
- [x] All plots are contour plots with outliers or pseudocolor plots.
- [x] A numerical value for number of cells or percentage (with statistics) is provided.

Methodology

Sample preparation

Lungs were perfused with 10 mL of PBS/2 mM EDTA via the right ventricle immediately after euthanasia. Lungs were processed with a gentleMACS dissociator (Miltenyi, lung program 1), and digested in 4 mL of RPMI-1640/5% HI-FBS containing 1 mg/mL collagenase D (Sigma-Aldrich) and 50 µg/mL DNase I (Sigma-Aldrich) for 30 min at 37 °C. Digested lung tissues were further dissociated with the gentleMACS dissociator (lung program 2) and then passed through a 70-µm cell strainer. Red blood cells were lysed with 3 mL ACK lysis buffer (Gibco) for 3 min and washed twice with RPMI-1640/5% HI-FBS.

Instrument

Cytek Aurora, Sony MAC900 cell sorter

Software

SpectroFlo software and Sony MAC900 cell sorter software was used for data collection. Data analysis was performed using FlowJo v10.8.1

Cell population abundance

Sorting of cell subsets was described in the method. Post-sort purity was determined by flow cytometry to be 80%~90%.

Gating strategy

Gating strategy can be found in supplemental Figure 1.

- [x] Tick this box to confirm that a figure exemplifying the gating strategy is provided in the Supplementary Information.
