# Supplementary material for: Impaired IFNγ responsiveness of monocyte-derived lung cells limits immunity to Mycobacterium tuberculosis
Source: Nat Commun. 2026 Jun 30;17:8131. doi: 10.1038/s41467-026-75096-4 (PMC13458731; doi:10.1038/s41467-026-75096-4)
Supplement: Supplementary file 3 [file 41467_2026_75096_MOESM3_ESM.pdf]

nature portfolio

Peer Review File

Impaired IFNγ responsiveness of monocyte-derived lung cells limits immunity to Mycobacterium tuberculosis

Corresponding Author: Dr Weihao Zheng

This file contains all reviewer reports in order by version, followed by all author rebuttals in order by version.

Version 0:

Reviewer comments:

Reviewer #1

(Remarks to the Author)
Summary
Zheng et al. investigate why monocyte-derived lung cells serve as a permissive niche for Mycobacterium tuberculosis during chronic infection. Using bulk RNA-seq, flow cytometry, and functional assays, they show that the CD11c^lo^ monocyte-derived subset (MNC1) expresses lower levels of IFNγ-signaling components (IFNGR2, STAT1, IRF1) and IFNγ-inducible genes such as CXCL9 and NOS2 than alveolar macrophages (AM) or CD11c^hi^ MNC2.
MNC1 cells have reduced STAT1 phosphorylation in response to exogenous IFNγ, diminished MHC-II expression, and a weaker ability to stimulate antigen-specific CD4 T cells. Type I IFN signaling suppresses IFNγ-driven MHC-II up-regulation in vitro and in mixed bone-marrow chimeras, and prior "contained" Mtb infection (CoMtb) enhances IFNγ responsiveness in monocyte-derived cells and improves bacterial control in these subsets in an IFNγ-dependent manner.
Overall, the study identifies heterogeneity in IFNγ responsiveness among lung mononuclear phagocytes as a mechanism that contributes to persistent Mtb infection and suggests a potential route to host-directed or vaccine strategies targeting monocyte-derived cells.

Major strengths
Clear mechanistic focus, built on strong prior work.
The manuscript logically extends previous observations that MNC1 are lysosome-poor and Mtb-permissive by interrogating IFNγ signaling at multiple levels (transcript, protein, functional readouts).
Multiple, complementary approaches.
The authors combine bulk RNA-seq, phospho-flow, immunofluorescence, antigen-presentation assays, in vitro BMDM differentiation, and in vivo bone-marrow chimera experiments, which converge on impaired IFNγ signaling as a key property of MNC1.
Integration of type I IFN and trained immunity.
Demonstrating that type I IFN dampens IFNγ-driven MHC-II and that CoMtb "trains" bone-marrow monocytes to respond better to IFNγ is conceptually appealing and links this work to broader discussions about detrimental type I IFN responses and trained immunity in TB.
Relevance to vaccine/host-directed therapy design.
The idea that improving IFNγ responsiveness specifically in monocyte-derived lung cells could enhance bacterial control is timely and is well articulated in the Discussion.
Major points / issues to address
1. Clarify the relative contributions of intrinsic programming vs ongoing type I IFN.
The data clearly show that MNC1 have reduced IFNGR2/STAT1/IRF1 expression and impaired pSTAT1 responses, but it remains somewhat unclear how much of this is "hard-wired" differentiation vs reversible suppression by type I IFN. It would strengthen the manuscript if the authors could:
• more explicitly address this point in the Discussion, and
• if available, include any data where type I IFN signaling is blocked (e.g. in Ifnar1^-/-^ MNC1) and IFNγ responsiveness (pSTAT1, MHC-II) is directly assessed.
2. Translational relevance to human lung macrophage subsets.
The authors appropriately acknowledge the limitation that all experiments are in mice and that the relevant subsets are rare in human lungs. This section could be expanded slightly by:
• briefly summarising any existing single-cell data on human lung monocyte-derived macrophages and IFNγ-responsive

gene signatures, and
• outlining concrete experimental approaches for testing whether similar heterogeneity exists in humans (e.g. BAL, resected lung tissue, ex vivo infection of monocyte-derived macrophages).
3. Strengthen the link between improved IFNγ responsiveness and bacterial control.
CoMtb reduces lung CFU and per-cell bacterial burden, and mixed chimeras show higher CFU in Ifngr1^-/-^ myeloid and MNC compartments. It would be useful if the authors could:
• provide more quantitative detail in the main text on effect sizes for CFU reduction at the subset level, and
• discuss alternative mechanisms by which CoMtb might alter monocyte-derived cell biology (beyond IFNγ responsiveness) and how their data support or argue against these possibilities.
4. Some figures could better highlight the key comparisons.
In several multi-panel figures (e.g. showing pSTAT1 and MHC-II across subsets), the relevant fold-change comparisons are described in the legend but are not immediately obvious. The clarity of the data presentation could be improved by:
• adding fold-change values or annotations directly on the graphs,
• standardising axis scales across subsets where appropriate, and
• ensuring colour schemes remain consistent for AM, MNC1, and MNC2 throughout.
5. Scope and novelty.
The work represents a mechanistically insightful but incremental advance, building on recent characterisations of lung macrophage heterogeneity in TB. It may help readers if the Introduction/Discussion more explicitly position this study relative to recent single-cell and functional papers, clearly delineating what is new here (e.g. identification of impaired IFNγ signaling pathway components specifically in MNC1 and their modulation by type I IFN and CoMtb).
Minor comments
• Ensure all acronyms are defined at first use in the main text and figures (e.g. MNC1/MNC2, CoMtb, BMDM).
• Check that each figure legend states the exact n, the statistical test used, and whether corrections for multiple comparisons were applied.
• Consider briefly stating in the Methods whether both sexes were used and, if so, whether any sex-specific differences were observed.
• A schematic summarising the proposed model (type I IFN → reduced IFNγ responsiveness/MHC-II in MNC1 vs trained monocytes in CoMtb → improved control) would be a helpful addition for readers who are less familiar with the various subsets.

Reviewer #2

(Remarks to the Author)
This manuscript examines the differential capacity of lung mononuclear phagocyte (MNP) subsets to control Mycobacterium tuberculosis (Mtb). They have previously shown that one of these subsets (MNC1s) were more permissive to Mtb viability, and here they show this same subset receiving significantly less IFNg signals than other subsets (alveolar macrophages (AM) and MNC2s) that control Mtb better. They also show that prior immunity mediated by contained Mtb (CoMtb model) increases IFNg signaling in MNPs including MNC1s and is associated with better Mtb control. These are potentially interesting and important findings, but some issues need to be addressed, as questions about some of the data need to be resolved before their conclusions can be fully justified.

Specific Comments:

1) In lines 81-82, it is stated that AM expressed more mRNA Ifngr2, Stat1, and Cxcl9 than MNC1 (Figure 1C), however, the data in the figure shows no significant difference in STAT1 and Cxcl9 expression.

2) A major concern is that in Figure 1D, and in many figures and supplemental figures throughout the paper, flow cytometry is used to show protein expression levels of components of the IFNg signaling pathways in the various MNP populations. Primary data is not shown, but it is depicted as bar graphs of MFI for the protein being interrogated. However, alveolar macrophages are highly autofluorescent and it is usually not possible draw conclusions that are simply based on MFI values of alveolar macrophages vs monocyte derived populations because they have very different autofluorescent baseline values in most if not all channels. When looking at mRNA expression in Figure 1C, alveolar macrophages seem to be most like MNC1s, and yet when looking at protein expression, as measured by flow cytometry MFI, they are quite different, raising concern that the findings depicted and Figure 1D and throughout the manuscript in other figures could be an artifact of AM autofluorescence. Controls need to be included, such as including staining with isotype control antibodies, and examples of primary flow plots need to be shown before this data can be accepted to support the conclusions stated.

3) Figure S7 shows that transfer of bone marrow cells from CoMtb mice improves the ability of the resulting chimeric mice to control Mtb upon subsequent challenge. In the Discussion, lines 248-256, say their results are consistent with trained immunity by bone marrow precursors of MNC1 and MNC2 cells. There are no details of how these experiments were performed in the Methods. These details should be concluded and also consideration of moving these results to a main figure since they are making important conclusions based on this supplemental figure. Were measures taken and controls performed to assure that Mtb itself was not inadvertently transferred to the recipients during the transfer of bone marrow cells? An alternative explanation of the data, if Mtb was transferred, is that it is not due to trained immunity, but rather, pre-existing immunity induced by the prior transfer of Mtb could have conferred the protection against subsequent challenge.

Reviewer #3

(Remarks to the Author)
The authors investigate how different myeloid cell populations respond to interferon signals during tuberculosis infection and make inferences on how these responses impact bacterial control. While interferon gamma is clearly a critical immune mediator of tuberculosis control, and therefore worth continued study, this manuscript provides little novel insight into the interplay between TB, myeloid cells, and interferon gamma. For example, interferon gamma signaling in AMs, monocytes, and monocyte-derived macrophages during tuberculosis infection has previously been examined (PMID: 32544188, 38236787, 38029747, and 40736456), which undermines statements like those in lines 50-51 about their being a lack of “understanding of myeloid subset-specific IFNg signaling during Mtb infection.” Additionally, type I interferon-mediated antagonism of interferon gamma signaling has been described in various infection contexts (some of which are cited in this manuscript), including during tuberculosis infection (PMID: 38029747), with the prior studies demonstrating reduced MHC II expression on monocyte-derived macrophages due to this antagonism. Finally, the coMtb experiments provide little novel insight, as the initial description of the coMtb model identified that coMtb induced interferon gamma, which upregulated MHC II expression on alveolar macrophages (PMID: 32673357). The coMtb-driven increase in MHC II expression has also been shown to occur in monocyte-derived macrophages in a recent study (PMID: 40736456). Thus, while the general concept under investigation in this manuscript is of interest to the field, this manuscript does not provide significant novel mechanistic understanding into interferon gamma responsiveness during TB infection.

Concerns:
The novelty of the manuscript is not clear as many of the conclusions rely on the unconventional MNC1 and MNC2 nomenclature. Limited phenotyping is performed comparing MNC1 and MNC2 cells and no effort is made to align their classification with standard monocyte-lineage cell classification. Based on the low CD11c expression of MNC1 cells, they are likely monocytes, while the MNC2 cells are monocyte-derived macrophages. This distinction could be easily established through additional phenotypic markers (e.g. MerTK, CD64, Ly6C, CCR2, CX3CR1). Considering monocytes differentiate into macrophages resulting in macrophages being more mature and inflammatory, many of the increases observed by MNC2 cells relative to MNC1 cells are not surprising. For example, upregulation of MHC II during monocyte maturation is a key marker of the “monocyte waterfall” concept established in 2012 (PMID: 22936024).

A foundational argument justifying the importance of examining the monocyte-derived macrophage’s inability to clear tuberculosis infection is the assertion that alveolar macrophages are better at controlling infection than monocyte-derived macrophages (lines 35-38). A more likely conclusion from the literature is that tuberculosis primarily resides in monocyte-derived macrophages not because of their inferior killing ability, but because of localization. Alveolar macrophages primarily reside in the alveoli of the lung, hence the name, while the bacteria transition into the lung parenchyma during the initial two weeks of infection, which is primarily occupied by monocyte-derived macrophages rather than alveolar macrophages. In fact, multiple groups have demonstrated that alveolar macrophage depletion improved tuberculosis control (PMID: 11254718 and 29500179), while depletion of interstitial macrophages or inhibition of monocyte recruitment by deficiency in CCR2 significantly elevated bacterial burden (PMID: 29500179 and 11438742). Thus, the text of the manuscript should be modified to align with the prevailing literature demonstrating a strong role for monocyte-derived macrophages rather than alveolar macrophages in tuberculosis control (although the response is insufficient to clear the infection!).

The representative flow cytometry gating scheme in Supplementary Fig. 1 doesn’t make sense. MNC are defined as CD11b+ cells with MHCII expression (the cutoff being 103 MHCII fluorescent intensity), but the bulk of the MNC1 population in the next gate has below 103 MHCII expression. Are the MNC cells just defined as CD11b+ cells that are then divided by CD11c expression or is MHCII used in the gating scheme?

In Fig. 1A and 1C, the MNC2 population clearly has more interferon gamma signaling than the AM or MNC1 population, but the results section avoids comparing the AM and MNC2 population (lines 71-87). It is important to display the statistically significant differences between the AM and MNC2 populations and discuss the analysis in the results (this statistical comparison is also missing in Figure 2 and multiple supplementary figures). Especially considering that a major argument of the paper is that AMs are better than MNCs at controlling TB due to AMs undergoing more interferon gamma signaling. This argument breaks down when examining the MNC2 population, as MNC2 cells harbor as many bacteria as the MNC1 population (Supp. Fig. 1B), even though they have more interferon gamma signaling than the AMs (Fig. 1A and 1C). Thus, the discussion should also be updated to include a comparison of the AM and MNC2 population and explain the disconnect between interferon gamma signaling and bacterial control in the MNC2 cells.

The authors state that IFNGR1 protein levels did not differ between AM, MNC1, and MNC2 cells (lines 84-86) but that is clearly not true, as AMs exhibited a statistically significant increase in IFNGR1 levels compared to MNC1 and likely MNC2 (for which the statistical analysis is not shown) in both experiments (Supp. Fig. 2). Additionally, Ifngr1 mRNA levels should be shown in Fig. 1C as type I interferon-driven reduction in interferon gamma responses by monocyte-derived macrophages has previously been attributed to lower levels of Ifngr1 expression during tuberculosis and listeria infections (PMID: 38029747 and 20123961).

The authors again misinterpret their results when discussing Supp. Fig. 3. They state that “the difference in IFNγ responsiveness among AM, MNC1, and MNC2 did not depend on the presence of Mtb in individual cells” (lines 100-102), but that is not true. There is no difference in pSTAT1 levels between bystander and infected cells for MNC1, but both AMs and MNC2 have statistically significantly higher pSTAT1 levels in infected cells than in bystander cells.

The data in Figure 4B demonstrates a ** significance between the control and IFNb groups but no changes are visible. This

is because there is logarithmic differences in MHCII MFI between the four conditions on the graph, but the data is presented on a linear scale. Thus, the graphs in Fig. 4B and 4C should by changed to log scale to properly display the data.

Figure 4 concludes that type I interferon negatively regulates interferon gamma-induced MHC II expression, and I agree that this phenomenon is clearly demonstrated in this figure. However, the in vivo data (Fig. 4E) demonstrates that cell type has a much larger impact (e.g. AM vs MNC1) on MHC II expression than the negative effect of type I interferon on interferon gamma-driven MHC II induction. This point needs to be more clearly addressed in the discussion.

In lines 242-244 of the discussion, the authors conclude that MNC1 cells harboring more bacteria than AM, MNC2 cells, or neutrophils are indicative of impaired IFNg responsiveness contributing to Mtb permissiveness. However, not all of the stated populations follow this rule, as the authors demonstrate in Figure 6H that AMs harbor the same amount of bacteria regardless of whether they express the interferon gamma receptor. Thus, the defective interferon gamma response of MNC1 cells may contribute to their poor TB control, but strong interferon gamma signaling by AMs does not prove it.

Version 1:

Reviewer comments:

Reviewer #1

(Remarks to the Author)
My questions were answered and I think this work should be published.

Reviewer #2

(Remarks to the Author)
The authors have adequately addressed my concerns.

I suggest the authors state in the manuscript that they plated the bone marrow on 7H11 plates and observed no growth (either Results or Methods section OK), in case readers have the same questions (the authors only stated this in the Response to reviewers, but not in the manuscript). An even better approach would have been to transfer bone marrow to uninfected mice to make sure infection cannot be detected in recipients. The stated argument that the Divangahi paper showed IV Mtb reversed trained immunity, may be due to dose (10^6 in that paper). A much lower dose would likely be transferred if bone marrow was infected and could have a different effect. Nevertheless, plating on 7H11 is acceptable to this reviewer and it is unlikely the bone marrow contained a small amount of Mtb that could grow upon in vivo transfer, but not culturable on 7H11 plates. Thus, I believe this response is adequate.

Reviewer #3

(Remarks to the Author)
While the authors have included additional statistical analysis and made minor text edits, it seems they did not provide any experimental data to address any reviewer concerns. Given significant concerns remain about the manuscript, this seems like a wasted opportunity. For example, questions about the lack of novelty for this manuscript remain and a clear mechanism underlying inadequate IFNg signaling in myeloid cells during TB infection is not presented. For example, the authors now state that there are mechanisms in addition to type I interferon that regulate IFNg responsiveness during Mtb infection. What are these mechanisms and how do they function during Mtb infection?
One of the mechanisms proposed for inadequate IFNg signaling during TB infection in this manuscript is downregulation of IFNg receptor, which is a well-established mechanism in TB and other diseases. The difference between the present study and previous work on subject is that this study suggests this defect is due to downregulation of IFNGR2 instead of IFNGR1. However, they do not provide an adequate explanation for why they do not recapitulate the reduction in IFNGR1 expression that has been observed across a number of studies examining type I interferon-mediated inhibition of IFNg signaling and do not provide a molecular mechanism for the negative regulation of IFNGR2 expression. Establishing a molecular mechanism that explains the reduction in IFNGR2 expression is critical given that impaired IFNg sensing is the major focus of this manuscript.

Regarding the novelty claims made by the authors, I cited multiple papers that directly assess and compare IFNg signaling across myeloid cell populations during TB infection in my initial review (e.g. PMID: 32544188, 38236787, 38029747, and 40736456). The authors address this lack of novelty by stating that “previous studies established the general importance of IFNg in these broad populations” but then make a novelty claim that they are the first to examine differences in the IFNg responsiveness across lung myeloid subsets. Perhaps the authors should re-read the manuscripts cited, as the manuscripts did look at IFNg signaling differences between lung myeloid populations thereby negating their novelty claim.

In response to my initial review stating pointing out their data argues that MNC2 actually have more IFNg signaling than AMs, the authors explained that they rank IFNg responsiveness as AM > MNC2 > MNC1. There arguments did not help clarify the matter as they then state “MNC2 cells express higher mRNA levels of IFNg pathway genes…compared to AM” in

the next sentence and even suggest that “MNC2 may be exposed to more IFNg than AM cells” later in the paragraph. Potentially, their hierarchy of IFNg signaling does not match their own mRNA expression data because of “some key genes” being higher in AM than MNC2 at the protein level, but they did not clarify which key genes. This is particularly confusing as they show in supplementary figure 5 that MHC II protein, a key IFNg regulated gene, is consistently higher in MNC2 cells than AMs at both evaluated time points of in vivo Mtb infection. Thus, their own data demonstrate that MNC2 cells undergo more IFNg signaling than AMs during in vivo Mtb infection. This key point undermines the argument that MNC poorly control TB infection relative to AMs due to less IFNg signaling, a major focus of the paper. The authors need to provide additional in vivo evidence for greater IFNg signaling in AMs compared to MNC2 cells if they want to make this argument. This is especially important as the majority of their data supporting their IFNg signaling hierarchy depends on ex vivo stims with IFNg. These ex vivo experiments measure what would happen if the cells sense IFNg, but does not measure whether the cells actual do sense IFNg in the lungs of infected mice, and therefore cannot establish the hierarchy of IFNg signaling in vivo during TB infection.

Version 2:

Reviewer comments:

Reviewer #2

(Remarks to the Author)
The authors have addressed my specific comment about including reference to the culture of the bone marrow cells in the Methods.

In my opinion, the authors have responded in a convincing manner to the issues raised by Reviewer #3. Regarding novelty, the summary of the findings of the cited manuscripts that the authors provided is quite accurate. Thus, while addressing IFNg signaling on different myeloid subsets more broadly, these other manuscripts do not address the specific findings of this paper, namely, that a specific subset of monocyte derived cells in the lung, a subset that is permissive to Mtb replication, is hyporesponsive to IFNg signaling. In addition, the authors have nicely clarified the distinction between the ability to respond to IFNg signals and whether those signals are actually being received, the latter of which likely depends on the microenvironment in the lung. Overall, I think the authors have adequately addressed the critiques of all the reviewers and that this manuscript will provide a good contribution to the literature.

Open Access This Peer Review File is licensed under a Creative Commons Attribution 4.0 International License, which permits use, sharing, adaptation, distribution and reproduction in any medium or format, as long as you give appropriate credit to the original author(s) and the source, provide a link to the Creative Commons license, and indicate if changes were made.
In cases where reviewers are anonymous, credit should be given to 'Anonymous Referee' and the source.
The images or other third party material in this Peer Review File are included in the article’s Creative Commons license, unless indicated otherwise in a credit line to the material. If material is not included in the article’s Creative Commons license and your intended use is not permitted by statutory regulation or exceeds the permitted use, you will need to obtain permission directly from the copyright holder.
To view a copy of this license, visit https://creativecommons.org/licenses/by/4.0/

We want to thank reviewers for their time in their critical appraisal of this work, and the helpful comments that they provided. We incorporated these comments into the manuscript. Our responses are below in black, and reviewers' comments are in blue. In the main manuscript, major adjustments are highlighted in yellow, while minor grammatical corrections and edits for brevity are not. We believe these changes have significantly improved the paper and again appreciate the reviewers' comments.

RESPONSES TO REVIEWER COMMENTS- NCOMMS-25-97684-T

Reviewer #1 (Remarks to the Author):

Summary

Zheng et al. investigate why monocyte-derived lung cells serve as a permissive niche for Mycobacterium tuberculosis during chronic infection. Using bulk RNA-seq, flow cytometry, and functional assays, they show that the CD11c^lo^ monocyte-derived subset (MNC1) expresses lower levels of IFNγ-signaling components (IFNGR2, STAT1, IRF1) and IFNγ-inducible genes such as CXCL9 and NOS2 than alveolar macrophages (AM) or CD11c^hi^ MNC2.

MNC1 cells have reduced STAT1 phosphorylation in response to exogenous IFNγ, diminished MHC-II expression, and a weaker ability to stimulate antigen-specific CD4 T cells. Type I IFN signaling suppresses IFNγ-driven MHC-II up-regulation in vitro and in mixed bone-marrow chimeras, and prior "contained" Mtb infection (CoMtb) enhances IFNγ responsiveness in monocyte-derived cells and improves bacterial control in these subsets in an IFNγ-dependent manner.

Overall, the study identifies heterogeneity in IFNγ responsiveness among lung mononuclear phagocytes as a mechanism that contributes to persistent Mtb infection and suggests a potential route to host-directed or vaccine strategies targeting monocyte-derived cells.

Major strengths

Clear mechanistic focus, built on strong prior work.

The manuscript logically extends previous observations that MNC1 are lysosome-poor and Mtb-permissive by interrogating IFNγ signaling at multiple levels (transcript, protein, functional readouts).

Multiple, complementary approaches.

The authors combine bulk RNA-seq, phospho-flow, immunofluorescence, antigen-presentation assays, in vitro BMDM differentiation, and in vivo bone-marrow chimera experiments, which converge on impaired IFNγ signaling as a key property of MNC1.

Integration of type I IFN and trained immunity.

Demonstrating that type I IFN dampens IFNγ-driven MHC-II and that CoMtb "trains" bone-marrow monocytes to respond better to IFNγ is conceptually appealing and links this work to broader discussions about detrimental type I IFN responses and trained immunity in TB.

Relevance to vaccine/host-directed therapy design.

The idea that improving IFNγ responsiveness specifically in monocyte-derived lung cells could enhance bacterial control is timely and is well articulated in the Discussion.

We thank the reviewer for appreciating the importance of our findings in this manuscript.

Major points / issues to address

1. Clarify the relative contributions of intrinsic programming vs ongoing type I IFN.

The data clearly show that MNC1 have reduced IFNGR2/STAT1/IRF1 expression and impaired pSTAT1 responses, but it remains somewhat unclear how much of this is "hard-wired" differentiation vs reversible suppression by type I IFN. It would strengthen the manuscript if the authors could:

• more explicitly address this point in the Discussion, and

• if available, include any data where type I IFN signaling is blocked (e.g. in Ifnar1^-/-^ MNC1) and IFNγ responsiveness (pSTAT1, MHC-II) is directly assessed.

We appreciate the insightful comments and suggestions. As shown in Figure 4E, the Ifnar1$^{-/-}$ subsets (MNC1, MNC2, and AM) show higher MHCII expression compared to the corresponding WT (Ifnar1$^{+/+}$) subsets, suggesting greater IFNγ responsiveness in the absence of Type-I IFN signaling. Although deletion of Ifnar1 fully restores IFNγ responsiveness in BMDM in vitro (Figure 4B), Ifnar1$^{-/-}$ MNC1 still show lower MHCII levels than Ifnar1$^{-/-}$ MNC2 and Ifnar1$^{-/-}$ AM, suggesting that a major portion of the differential IFNγ responsiveness in different MNP subsets in vivo is attributable to Type-I IFN, but that one or more additional mechanisms contribute. activity. We have added more comments for this point in the Discussion, read as "Interestingly, IFNγ responsiveness in MNC1 is not fully restored by deletion of Ifnar1, suggesting that other mechanisms contribute to regulating IFNγ responsiveness in lung MNC subsets in vivo during Mtb infection." -lines 270-272

2. Translational relevance to human lung macrophage subsets.

The authors appropriately acknowledge the limitation that all experiments are in mice and that the relevant subsets are rare in human lungs. This section could be expanded slightly by:

• briefly summarising any existing single-cell data on human lung monocyte-derived macrophages and IFNγ-responsive gene signatures, and

• outlining concrete experimental approaches for testing whether similar heterogeneity exists in humans (e.g. BAL, resected lung tissue, ex vivo infection of monocyte-derived macrophages).

Thank you for these suggestions. We have mined results from single-cell studies of human TB tissues and have added additional comments in the Discussion. Lines 302-309, "Recent single-cell sequencing studies have revealed heterogeneity of lung monocyte-derived macrophages in human TB[52–54], and a potential difference in IFNγ responses among macrophage subsets in TB granulomas[52]. However, functional studies are required to characterize IFNγ responsiveness in live macrophage subsets isolated from resected lung tissues of human TB patients. These are limited by the rarity of appropriate human samples in proximity to facilities for the procedures required, and considerable dedicated effort will be required to determine which of our findings are applicable to humans."

3. Strengthen the link between improved IFNγ responsiveness and bacterial control.
CoMtb reduces lung CFU and per-cell bacterial burden, and mixed chimeras show higher CFU in Ifngr1^-/-^ myeloid and MNC compartments. It would be useful if the authors could:

• provide more quantitative detail in the main text on effect sizes for CFU reduction at the subset level, and

• discuss alternative mechanisms by which CoMtb might alter monocyte-derived cell biology (beyond IFNγ responsiveness) and how their data support or argue against these possibilities.

We appreciate these suggestions and have provided the fold change for CFU reduction at the subset level for CoMtb vs control in the main text (lines 197-199), read as "Moreover, we found that CoMtb

reduced Mtb burdens in AM (1.2-fold decrease), neutrophils (1.5-fold decrease), and especially in MNC1 (2.7-fold decrease) and MNC2 (1.8-fold decrease)."

We agree with reviewer #1 that CoMtb may alter monocyte-derived cell biology via alternative mechanisms beyond IFNγ responsiveness. We have added more comments in the Discussion, lines 289-291: However, we cannot exclude the possibility that CoMtb may alter other cell-intrinsic mechanisms, such as lysosome biogenesis and autophagy, to synergize with improved IFNγ responsiveness and enhance bacterial control, which warrants further study.

4. Some figures could better highlight the key comparisons.

In several multi-panel figures (e.g. showing pSTAT1 and MHC-II across subsets), the relevant fold-change comparisons are described in the legend but are not immediately obvious. The clarity of the data presentation could be improved by:

• adding fold-change values or annotations directly on the graphs,
•standardising axis scales across subsets where appropriate, and
• ensuring colour schemes remain consistent for AM, MNC1, and MNC2 throughout.

Thank you for these suggestions. We have added fold-change values for Figures 1-3. We also standardized the Y-axis scales for subsets where appropriate (Figure 1D; Figure 2B,2D,2E; Figure 4B-4C; Figure 5D; Figure 6J). Lastly, we have also adjusted the color schemes to ensure consistency across AM, MNC1, and MNC2 in Figures 1-3.

5. Scope and novelty.

The work represents a mechanistically insightful but incremental advance, building on recent characterisations of lung macrophage heterogeneity in TB. It may help readers if the Introduction/Discussion more explicitly position this study relative to recent single-cell and functional papers, clearly delineating what is new here (e.g. identification of impaired IFNγ signaling pathway components specifically in MNC1 and their modulation by type I IFN and CoMtb).

We thank the reviewer for this constructive suggestion. Single-cell data from our own and other studies have mapped lung MNP heterogeneity at high resolution (PMID: 39813329, 34292313, 38029747); however, we know little about the mechanisms of functional heterogeneity that support Mtb survival in distinct lung MNP subsets. Specifically, it was not previously known whether distinct lung MNP subsets differ in their responsiveness to IFNγ during primary Mtb infection, and whether interventions can alter IFNγ responsiveness in these subsets. We have revised the Introduction (lines 43-44, 54-56, 64-69, 72-81) and Discussion (lines 218-227, 241-275) to explicitly delineate our novel contributions:

(1) Identification of the bottleneck in IFNγ signalling: While our previous work revealed that monocyte-derived cells (MNCs) are permissive niches during chronic infection, our present work identifies subset differences in IFNγ responsiveness and the specific failure of IFNγ signaling pathway components within the MNC1 subset (the subset that contains the largest number of viable Mtb per cell; PMID 38701094). Specifically, we demonstrate that MNC1 cells possess a lower expression of critical signaling nodes (e.g., IFNGR2, STAT1, IRF1) compared to their MNC2 counterparts and AM, rendering them functionally less responsive to IFNγ.

(2) Mechanistic Crosstalk (Type I vs. Type II IFN): We now more clearly highlight the discovery that Type I IFN activity suppresses IFNγ-driven MHC-II expression in MNC1 cells. This provides a mechanistic link between the known detrimental role of Type I IFN in TB and the failure of CD4 T-cell-mediated control at the cellular level.

(3) Modulation by CoMtb: Using the Contained Mtb (CoMtb) model, we demonstrate for the first time that state of impaired IFNγ responsiveness is not exclusively an inherent terminal differentiation trait of MNC1, but is modifiable. We found that CoMtb-induced systemic changes reprogram these cells to overcome the signaling blockade, providing the entry point to identifying targets for host-directed therapy.

(4) Inform the design of new TB vaccines. TB vaccines that predominantly induce IFNγ responses have shown limited efficacy (PMID: 30001523). In addition, murine studies indicate that increasing IFNγ production does not enhance Mtb control in the lungs (PMID: 27244558). Together, results have been interpreted as evidence that effective TB immunity requires other T cell effector mechanisms. While we agree with that interpretation, the results are also consistent with the interpretation that IFNγ action is limited in TB. Our work provides support for the latter model and provides mechanistic insight into these results.

Minor comments

• Ensure all acronyms are defined at first use in the main text and figures (e.g. MNC1/MNC2, CoMtb, BMDM).

• Check that each figure legend states the exact n, the statistical test used, and whether corrections for multiple comparisons were applied.

• Consider briefly stating in the Methods whether both sexes were used and, if so, whether any sex-specific differences were observed.

• A schematic summarising the proposed model (type I IFN → reduced IFNγ responsiveness/MHC-II in MNC1 vs trained monocytes in CoMtb → improved control) would be a helpful addition for readers who are less familiar with the various subsets.

We appreciate reviewer#1's careful reading and suggestions. We have double-checked the manuscript to ensure that all acronyms are defined at first use in the main text and figures.

We have also updated figure legends to state the exact n, the statistical test used, and corrections for multiple comparisons applied.

Both sexes were used in this study. Extensive experiments and projects in our lab demonstrate no sex-specific differences in our murine TB models. Therefore, sex was not a consideration in this study; sample sex information has not been collected. We have updated this in the Methods section (lines 318-321) and the Reporting Summary as required by the journal.

In addition, we have added a schematic summarizing this work as suggested (see Figure 7).

Reviewer #2 (Remarks to the Author):

This manuscript examines the differential capacity of lung mononuclear phagocyte (MNP) subsets to control Mycobacterium tuberculosis (Mtb). They have previously shown that one of these subsets (MNC1s) were more permissive to Mtb viability, and here they show this same subset receiving significantly less IFNg signals than other subsets (alveolar macrophages (AM) and MNC2s) that control Mtb better. They also show that prior immunity mediated by contained Mtb (CoMtb model) increases IFNg signaling in MNPs including MNC1s and is associated with better Mtb control. These are potentially interesting and important findings, but some issues need to be addressed, as questions about some of the data need to be resolved before their conclusions can be fully justified.
Thank you for appreciating the importance of our findings in this work.

Specific Comments:

1) In lines 81-82, it is stated that AM expressed more mRNA Ifngr2, Stat1, and Cxcl9 than MNC1 (Figure 1C), however, the data in the figure shows no significant difference in STAT1 and Cxcl9 expression.
We appreciate this comment and apologize for the confusion. We have modified this sentence for clarity and moved Ifngr1 data to Figure 1 as suggested by reviewer#3, read as "This revealed that MNC1 had lower mRNA expression of *Ifngr1, Ifngr2*, *Stat1*, *Irf1*, *Cxcl9*, and *Nos2* compared to MNC2, while AM expressed significantly more *Ifngr2* mRNA than MNC1, with a similar but non-significant trend observed for *Ifngr1, Stat1*, and *Cxcl9* (Figure 1C)." (Lines 94-97).

2) A major concern is that in Figure 1D, and in many figures and supplemental figures throughout the paper, flow cytometry is used to show protein expression levels of components of the IFNg signaling pathways in the various MNP populations. Primary data is not shown, but it is depicted as bar graphs of MFI for the protein being interrogated. However, alveolar macrophages are highly autofluorescent and it is usually not possible draw conclusions that are simply based on MFI values of alveolar macrophages vs monocyte derived populations because they have very different autofluorescent baseline values in most if not all channels. When looking at mRNA expression in Figure 1C, alveolar macrophages seem to be most like MNC1s, and yet when looking at protein expression, as measured by flow cytometry MFI, they are quite different, raising concern that the findings depicted and Figure 1D and throughout the manuscript in other figures could be an artifact of AM autofluorescence. Controls need to be included, such as including staining with isotype control antibodies, and examples of primary flow plots need to be shown before this data can be accepted to support the conclusions stated.
Thanks for pointing this out, and we agree that alveolar macrophages and monocyte-derived cells have different autofluorescence in some flow channels. We noticed that cell autofluorescence can confound results when antigens are detected at lower levels. Therefore, we have double checked that all MFI data for the lung subsets are background-subtracted using the FMO control or uninfected control to ensure that the observed differences are real, and we updated Figure 6C and 6J. The updated Figure 6C shows that deleting the IFNγ receptor slightly increased bacterial burden in AM from coMtb mice (Figure 6J). Moreover, the impact of IFNγR deletion remains significantly more pronounced in monocyte-derived cells regarding bacterial control (Figure 6J-6K). This does not alter the conclusion that CoMtb plays a minimal role in AM at the later stage of Mtb infection. We have added representative histograms of proteins analyzed by flow cytometry for lung MNP subsets as suggested (see Figure S2). We also added details

about MFI quantification in the "Quantification and statistical analysis" section, read as "MFI data for the lung subsets are background-subtracted using the fluorescence minus one (FMO) control or uninfected control." (Lines 414-415)

3) Figure S7 shows that transfer of bone marrow cells from CoMtb mice improves the ability of the resulting chimeric mice to control Mtb upon subsequent challenge. In the Discussion, lines 248-256, say their results are consistent with trained immunity by bone marrow precursors of MNC1 and MNC2 cells. There are no details of how these experiments were performed in the Methods. These details should be concluded and also consideration of moving these results to a main figure since they are making important conclusions based on this supplemental figure. Were measures taken and controls performed to assure that Mtb itself was not inadvertently transferred to the recipients during the transfer of bone marrow cells? An alternative explanation of the data, if Mtb was transferred, is that it is not due to trained immunity, but rather, pre-existing immunity induced by the prior transfer of Mtb could have conferred the protection against subsequent challenge.

We regret the oversight and appreciate these suggestions and comments. We have added more details for the chimera experiment using bone marrow cells from CoMtb mice in "Generation of bone marrow chimeras" section (see lines 400-401). We have also moved the results from Figure S7 to Figure 6G-6H as suggested.

For the bone marrow cells from CoMtb mice, we plated the bone marrow cells on 7H11 plates and did not observe colonies after 3-4 weeks of incubation at 37°C, suggesting that no Mtb was transferred, which is consistent with the previous study demonstrating that Mtb is restricted to the draining lymph node without dissemination to other organs in the CoMtb model (PMID: 32673357). In addition, a previous study (PMID: 33125891) has shown that intravenous Mtb infection impairs trained immunity. Therefore, if Mtb were transferred via blood, it would not be expected to induce protective immunity as observed in this manuscript.

Reviewer #3 (Remarks to the Author):

The authors investigate how different myeloid cell populations respond to interferon signals during tuberculosis infection and make inferences on how these responses impact bacterial control. While interferon gamma is clearly a critical immune mediator of tuberculosis control, and therefore worth continued study, this manuscript provides little novel insight into the interplay between TB, myeloid cells, and interferon gamma. For example, interferon gamma signaling in AMs, monocytes, and monocyte-derived macrophages during tuberculosis infection has previously been examined (PMID: 32544188, 38236787, 38029747, and 40736456), which undermines statements like those in lines 50-51 about their being a lack of "understanding of myeloid subset-specific IFNg signaling during Mtb infection." Additionally, type I interferon-mediated antagonism of interferon gamma signaling has been described in various infection contexts (some of which are cited in this manuscript), including during tuberculosis infection (PMID: 38029747), with the prior studies demonstrating reduced MHC II expression on monocyte-derived macrophages due to this antagonism. Finally, the coMtb experiments provide little novel insight, as the initial description of the coMtb model identified that coMtb induced interferon gamma, which upregulated MHC II expression on alveolar macrophages (PMID: 32673357). The coMtb-driven increase in MHC II expression has also been shown to occur in monocyte-derived macrophages in a recent study (PMID: 40736456). Thus, while the general concept under investigation in this manuscript is of interest to the field, this manuscript does not provide significant novel mechanistic understanding into interferon gamma responsiveness during TB infection.

We thank the reviewer for these comments and for recognizing the importance of this work. Our study provides a highly granular, subset-specific mechanism that explains a long-standing paradox in TB immunology: why Mtb persists in the lung despite the presence of high IFNγ levels.

We agree that previous studies established the general importance of IFNγ in these broad populations; however, our work is the first to examine differences in IFNγ responsiveness across lung mononuclear subsets. We demonstrate that MNC1 cells, the cell subset that we found harbors the largest number of live Mtb per infected cell (PMID: 38701094), uniquely express lower levels of signaling proteins in the IFNγ response pathway and are hyporesponsive to IFNγ, making them a more favorable niche for Mtb. This provides novel mechanistic insight into functional heterogeneity within lung mononuclear phagocyte subsets, and provides functional evidence why Mtb persists even in an IFNγ-rich lung environment, which previous studies did not fully address. In addition, we provide new mechanistic insights into deficient IFNγ signaling in MNC1 cells: we found that defective signaling occurs at a proximal step (tyrosine phosphorylation of STAT1) and that this is likely secondary to deficient expression of IFNGR2, the signaling subunit of IFNγ receptors. While little is known regarding the regulation of IFNGR2 expression, our findings reveal a signaling step to pursue with host-directed therapy discovery efforts.

While type I IFN is known to antagonize certain responses to IFNγ, our study provides a novel mechanistic link by showing it contributes to MHC-II downregulation in the MNC1 subset, directly impairing CD4 T cell activation in a way not previously characterized. Importantly, $Ifnar1^{-/-}$ MNC1 still show a lower MHCII level than $Ifnar1^{-/-}$ MNC2 and $Ifnar1^{-/-}$ AM, suggesting that Type-I IFN signaling partially regulates IFNγ responsiveness in different MNP subsets in vivo, and that additional mechanisms may regulate IFNγ responsiveness in these cell subsets.

The reviewer correctly notes that the coMtb model induces IFNγ signaling and increases MHC-II. However, our study reveals novel mechanisms by demonstrating that prior immunity can reprogram monocyte-derived cells to restore IFNγ responsiveness and that IFNγR signaling is required for coMTB-mediated protection. This suggests that the immune system's failure during primary infection is not just

a lack of IFNγ, but a failure of specific cells to respond to it—a defect that can be corrected by immunity provided by prior exposure to a low-level, contained infection. This "restoration of response" provides a novel conceptual framework for TB vaccine design.
We have cited the papers the reviewer mentioned and revised the Introduction (lines 43-44, 54-56, 64-69, 72-81) and Discussion (lines 218-227, 241-275) to explicitly delineate our novel contributions.

Concerns:
The novelty of the manuscript is not clear as many of the conclusions rely on the unconventional MNC1 and MNC2 nomenclature. Limited phenotyping is performed comparing MNC1 and MNC2 cells and no effort is made to align their classification with standard monocyte-lineage cell classification. Based on the low CD11c expression of MNC1 cells, they are likely monocytes, while the MNC2 cells are monocyte-derived macrophages. This distinction could be easily established through additional phenotypic markers (e.g. MerTK, CD64, Ly6C, CCR2, CX3CR1). Considering monocytes differentiate into macrophages resulting in macrophages being more mature and inflammatory, many of the increases observed by MNC2 cells relative to MNC1 cells are not surprising. For example, upregulation of MHC II during monocyte maturation is a key marker of the "monocyte waterfall" concept established in 2012 (PMID: 22936024).
We thank the reviewer for the opportunity to clarify our nomenclature. The similar gating strategy for monocyte-derive cell (MNC) subsets $CD11c^{lo}$ MNC1 and $CD11c^{hi}$ MNC2 has been widely used in TB publications from our group and others (PMID: 38701094, 39813329, 32544188, 30365557, 35695454, 17675513, 22264515, 21555529, 41171885), and were formerly termed recruited macrophages and monocyte-derived dendritic cells, respectively. Although increasing evidence from others and ours suggests that these two subsets are macrophage-like cells (PMID: 38701094, 39813329, 32544188), we and others found that MerTK and CD64 are inadequate markers for monocyte-derived cells (MNC) in the context of Mtb infection (PMID: 38701094, 32544188). Therefore, we continue to use MNC1 and MNC2 for MNC subsets in this study, as we did in our recent work (PMID: 38701094, 39813329, 32544188, 41171885). We find that attempting to adhere to the oft-used term of 'interstitial macrophages' overlooks the evidence that the vast majority of monocytic cells in the lungs of Mtb-infected mice are newly recruited and therefore distinct from resident interstitial macrophages in the lungs. We appreciate the shortcomings of the MNC1/MNC2 nomenclature and will be happy to contribute to future efforts to arrive at a consensus terminology. In addition, the novelty of our study lies not in the classification itself, but in the discovery of functional differences in IFNγ responsiveness across subsets and in the finding that Mtb exploits these differences to promote persistence.
We agree that MNC1 and MNC2 may represent different stages of monocyte differentiation. However, using MerTK and CD64 to gate MerTK+CD64+ cells in the MNC1 and MNC2 subsets, we found that MerTK+CD64+ MNC1 express lower levels of MHCII than AM and MerTK+CD64+ MNC2 (see below, Figure R1), suggesting that the 'monocyte waterfall' concept does not apply to the TB context. Moreover, we also find that limiting analyses to gates defined by MerTK and/or CD64 excludes cells of interest, including cells that harbor fluorescent protein-expressing Mtb.

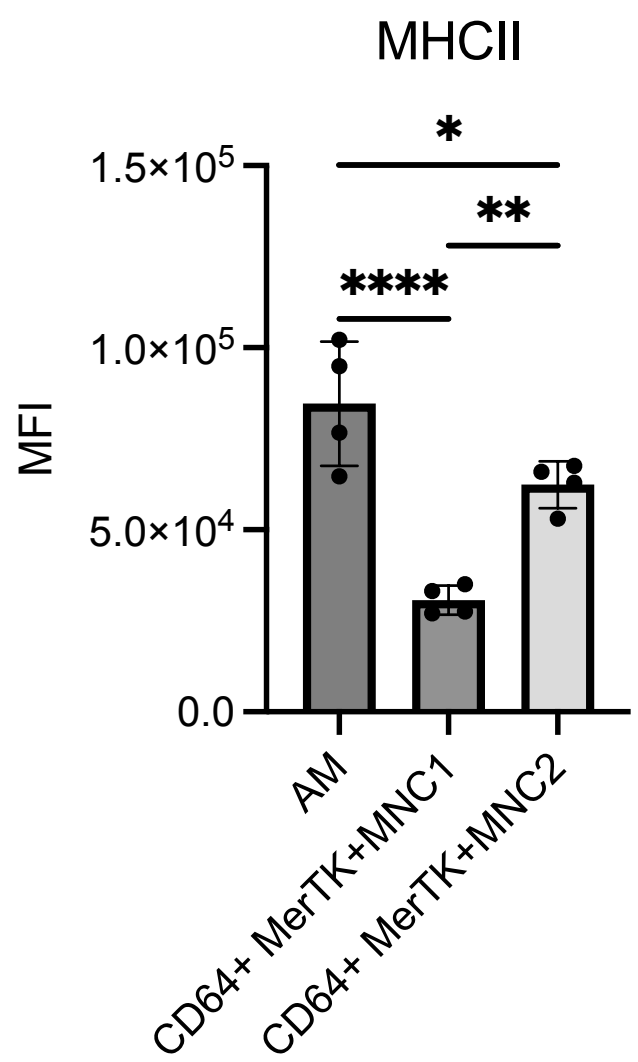

Figure R1. MHC-II expression level in AM, MerTK+CD64+ MNC1, and MerTK+CD64+ MNC2. C57BL/6 mice were infected with Mtb H37Rv for 28 days. Lung cells were harvested for flow cytometry analysis. Results are presented as mean ± SD. One-way ANOVA with Tukey's multiple comparison test was used to determine statistical significance. *p<0.05, **p<0.01, ****p<0.0001.

A foundational argument justifying the importance of examining the monocyte-derived macrophage's inability to clear tuberculosis infection is the assertion that alveolar macrophages are better at controlling infection than monocyte-derived macrophages (lines 35-38). A more likely conclusion from the literature is that tuberculosis primarily resides in monocyte-derived macrophages not because of their inferior killing ability, but because of localization. Alveolar macrophages primarily reside in the alveoli of the lung, hence the name, while the bacteria transition into the lung parenchyma during the initial two weeks of infection, which is primarily occupied by monocyte-derived macrophages rather than alveolar macrophages. In fact, multiple groups have demonstrated that alveolar macrophage depletion improved tuberculosis control (PMID: 11254718 and 29500179), while depletion of interstitial macrophages or inhibition of monocyte recruitment by deficiency in CCR2 significantly elevated bacterial burden (PMID: 29500179 and 11438742). Thus, the text of the manuscript should be modified to align with the prevailing literature demonstrating a strong role for monocyte-derived macrophages rather than alveolar macrophages in tuberculosis control (although the response is insufficient to clear the infection!).

We appreciate this comment and suggestion. Indeed, previous studies have used depletion experiments to show that, before the initiation of T cell responses, alveolar macrophages support Mtb proliferation, whereas interstitial macrophages are relatively resistant. While CCR2 is required for monocyte recruitment, we previously showed that it is also required for the development of T cell responses during Mtb infection (PMID: 11438742), making it challenging to disentangle the contributions of monocyte-derived cells and impaired T cell responses to lung Mtb control. In contrast, transgenic mice overexpressing CCL2, the CCR2 ligand, were more susceptible to tuberculosis (PMID: 7594486). Nevertheless, our group and others found that after the development of T cell responses (PMID: 38701094, 39813329, 32544188, 38977711, 20389020), the infection stage we focus on in this study, monocyte-derived cells are permissive to Mtb, while alveolar macrophages restrict Mtb.

We agree with the reviewer that monocyte-derived cells represent an important target for TB therapy, as they are the major Mtb reservoirs during chronic infection. Our current study provides a novel mechanism

for why T cell responses are insufficient to clear the infection in these cells. We have followed the reviewer's suggestion to modify the text to include these points (lines 250-260).

The representative flow cytometry gating scheme in Supplementary Fig. 1 doesn't make sense. MNC are defined as CD11b+ cells with MHCII expression (the cutoff being 103 MHCII fluorescent intensity), but the bulk of the MNC1 population in the next gate has below 103 MHCII expression. Are the MNC cells just defined as CD11b+ cells that are then divided by CD11c expression or is MHCII used in the gating scheme?
We thank the reviewer for this question. MNC cells are defined as CD11b+ MHCII+ cells in Figure S1A. In the next flow plot after MNC gate, CD11c is mainly used to define MNC subsets: MNC1 are $CD11c^{lo}$, and MNC2 are $CD11c^{hi}$. MHCII is used to visualize the distribution of MNC1/MNC2 and the pattern of MHCII expression. Therefore, the gate below 10^3 MHCII expression will help ensure that all MNC1 cells are included, as all MNC are MHCII+. However, to avoid confusion, we have moved the gate to 10^3 for MHCII expression in MNC1 and updated the last flow plot in Figure S1A.

In Fig. 1A and 1C, the MNC2 population clearly has more interferon gamma signaling than the AM or MNC1 population, but the results section avoids comparing the AM and MNC2 population (lines 71-87). It is important to display the statistically significant differences between the AM and MNC2 populations and discuss the analysis in the results (this statistical comparison is also missing in Figure 2 and multiple supplementary figures). Especially considering that a major argument of the paper is that AMs are better than MNCs at controlling TB due to AMs undergoing more interferon gamma signaling. This argument breaks down when examining the MNC2 population, as MNC2 cells harbor as many bacteria as the MNC1 population (Supp. Fig. 1B), even though they have more interferon gamma signaling than the AMs (Fig. 1A and 1C). Thus, the discussion should also be updated to include a comparison of the AM and MNC2 population and explain the disconnect between interferon gamma signaling and bacterial control in the MNC2 cells.
We appreciate this helpful suggestion and have included a comparison of the AM and MNC2 populations in Figures 1-3, S3-S5 and added more comments in the Discussion (lines 266-269). Additionally, we can clarify several points.
First, using different methods (CFU assays on sorted cell subsets, Live/dead reporter strains and single-cell microscopic quantitation, and intensity of intracellular Mtb fluorescence by flow cytometry), our previous study revealed that among infected subsets, MNC1 harbors more live Mtb per cell than AM and MNC2, and MNC2 harbors an intermediate level of bacterial burden (PMID: 38701094; also see Figure 6F, control group).
Second, our current work revealed differences in IFNγ responsiveness among AM, MNC1, and MNC2: AM > MNC2 > MNC1. Although MNC2 cells express higher mRNA levels of IFNγ pathway genes, at the protein level, some of these key genes are expressed at lower levels in MNC2 compared to AM. This suggests that MNC2 may be exposed to more IFNγ than AM cells, possibly due to their localization. However, MNC2 show a greater IFN-I signaling than AM, which may antagonize IFNγ signaling at a posttranscriptional level despite exposure to more IFNγ. This also holds true for infected cell subsets (Figure S3). Therefore, bacterial control ability is associated with IFNγ responsiveness among AM, MNC1, and MNC2 (Figures 1-4), and the CoMtb-mediated restoration of IFNγ responsiveness in MNC cells improves bacterial control within these subsets (Figures 5-6).

The authors state that IFNGR1 protein levels did not differ between AM, MNC1, and MNC2 cells (lines 84-86) but that is clearly not true, as AMs exhibited a statistically significant increase in IFNGR1 levels compared to MNC1 and likely MNC2 (for which the statistical analysis is not shown) in both experiments (Supp. Fig. 2). Additionally, Ifngr1 mRNA levels should be shown in Fig. 1C as type I interferon-driven reduction in interferon gamma responses by monocyte-derived macrophages has previously been attributed to lower levels of Ifngr1 expression during tuberculosis and listeria infections (PMID: 38029747 and 20123961).

We thank the reviewer for this suggestion and have added plots of IFNGR1 mRNA and protein levels in Figure 1. We also modified the sentence to avoid confusion- Lines 99-101: "Although AM expressed more IFNGR1 protein than MNC1, the protein level of IFNGR1 did not differ between MNC1 and MNC2 (Figure 1D and Figure S2)."

The authors again misinterpret their results when discussing Supp. Fig. 3. They state that "the difference in IFNγ responsiveness among AM, MNC1, and MNC2 did not depend on the presence of Mtb in individual cells" (lines 100-102), but that is not true. There is no difference in pSTAT1 levels between bystander and infected cells for MNC1, but both AMs and MNC2 have statistically significantly higher pSTAT1 levels in infected cells than in bystander cells.

We appreciate reviewer's careful reading and regret the confusion. We observe similar differences in IFNγ responsiveness within the individual cell subsets (AM, MNC1, and MNC2), regardless of the presence of intracellular bacteria in the cells comprising these subsets. We have modified the sentence for clarity, read as "Interestingly, although infected AM and infected MNC2 responded to IFNγ better than their bystander counterparts, the subset difference in IFNγ responsiveness between AM, MNC1, and MNC2 did not depend on the presence of Mtb in individual cells (Figure S3)."(lines 115-118)

The data in Figure 4B demonstrates a ** significance between the control and IFNb groups but no changes are visible. This is because there is logarithmic differences in MHCII MFI between the four conditions on the graph, but the data is presented on a linear scale. Thus, the graphs in Fig. 4B and 4C should by changed to log scale to properly display the data.

Thank you for this suggestion. We have changed linear scale to log scale for Figure 4B and 4C.

Figure 4 concludes that type I interferon negatively regulates interferon gamma-induced MHC II expression, and I agree that this phenomenon is clearly demonstrated in this figure. However, the in vivo data (Fig. 4E) demonstrates that cell type has a much larger impact (e.g. AM vs MNC1) on MHC II expression than the negative effect of type I interferon on interferon gamma-driven MHC II induction. This point needs to be more clearly addressed in the discussion.

We appreciate this insightful comment. We agree that type I interferon partially suppresses IFNγ-induced MHC II expression in vivo, and cell type has a larger impact, suggesting that cell differentiation mechanisms may regulate IFNγ responsiveness within lung MNP subsets in vivo during Mtb infection. We have added more comments for this point in the Discussion (lines 270-272), read as "Interestingly, IFNγ responsiveness in MNC1 is not fully restored by deletion of *Ifnar1*, suggesting that other mechanisms may contribute to regulating IFNγ responsiveness in lung MNC subsets in vivo during Mtb infection."

In lines 242-244 of the discussion, the authors conclude that MNC1 cells harboring more bacteria than AM, MNC2 cells, or neutrophils are indicative of impaired IFNg responsiveness contributing to Mtb permissiveness. However, not all of the stated populations follow this rule, as the authors demonstrate in Figure 6H that AMs harbor the same amount of bacteria regardless of whether they express the interferon gamma receptor. Thus, the defective interferon gamma response of MNC1 cells may contribute to their poor TB control, but strong interferon gamma signaling by AMs does not prove it.

We thank the reviewer for this insightful comment. In accordance with the suggestion from Reviewer #2, we have re-analyzed our MFI data by subtracting the background using the appropriate control group MFI. Consequently, Figure 6H has been updated (now Figure 6J in the revised version). The updated results show that deleting the IFNγ receptor slightly increased bacterial burden in AM from coMtb mice (Figure 6J). Moreover, the impact of IFNγR deletion remains significantly more pronounced in monocyte-derived cells regarding bacterial control (Figure 6J-6K). These are consistent with results in Figure 5D-5G and Figure 6F, which showed that CoMtb cannot further enhance IFNγ function in AM and has a limited effect on reducing bacterial burden in AM compared to MNC subsets. Our results support the model that while IFNγ signaling is broadly required for bacterial control, its relative importance varies between cell types at different stages of infection.

These findings indicate that the mechanisms of enhanced immunity in CoMtb mice are cell-type specific. MNC1 cells face a critical 'bottleneck' due to impaired IFNγ signaling during primary infection; thus, restoring this responsiveness via CoMtb is a primary driver of their improved control. In contrast, while IFNγR signaling contributes to AM function at early stages (PMID: 32673357, 38236787, 41171885), it plays a more limited role in the CoMtb-mediated protection of AMs during the later chronic stage of infection.

We have clarified this distinction in the revised Discussion (lines 280-289).

Additionally, we have updated the 'Quantification and statistical analysis' section to specify: “MFI data for the lung subsets are background-subtracted using the fluorescence minus one (FMO) control or uninfected control.” (Lines 414-415)

We thank the reviewers again for their time in reviewing our revised manuscript. We are glad that our revisions have addressed all concerns from reviewers #1 and #2. We appreciate the reviewer #2's further textual suggestion and reviewer #3's new constructive comments. We have made changes in the manuscript as suggested. Reviewer comments are in blue, and our responses are in black. We believe these changes have improved the paper and again appreciate the reviewers' input.

REVIEWER COMMENTS

Reviewer #1 (Remarks to the Author):

My questions were answered and I think this work should be published.

We are grateful for the positive assessment and support for publication.

Reviewer #2 (Remarks to the Author):

The authors have adequately addressed my concerns.

I suggest the authors state in the manuscript that they plated the bone marrow on 7H11 plates and observed no growth (either Results or Methods section OK), in case readers have the same questions (the authors only stated this in the Response to reviewers, but not in the manuscript). An even better approach would have been to transfer bone marrow to uninfected mice to make sure infection cannot be detected in recipients. The stated argument that the Divangahi paper showed IV Mtb reversed trained immunity, may be due to dose (10^6 in that paper). A much lower dose would likely be transferred if bone marrow was infected and could have a different effect. Nevertheless, plating on 7H11 is acceptable to this reviewer and it is unlikely the bone marrow contained a small amount of Mtb that could grow upon in vivo transfer, but not culturable on 7H11 plates. Thus, I believe this response is adequate.

We are pleased that our revisions have addressed all concerns and appreciate this suggestion. We have added "$5x10^6$ bone marrow cells in triplicate were plated on 7H11 agar plates and no Mtb growth was observed after 3-4 weeks of incubation at 37°C" (lines 419-421) to the Method section "Generation of bone marrow chimeras".

Reviewer #3 (Remarks to the Author):

While the authors have included additional statistical analysis and made minor text edits, it seems they did not provide any experimental data to address any reviewer concerns. Given significant concerns remain about the manuscript, this seems like a wasted opportunity. For example, questions about the lack of novelty for this manuscript remain and a clear mechanism underlying inadequate IFNg signaling in myeloid cells during TB infection is not presented. For example, the authors now state that there are mechanisms in addition to type I interferon that regulate IFNg responsiveness during Mtb infection. What are these mechanisms and how do they function during Mtb infection?
One of the mechanisms proposed for inadequate IFNg signaling during TB infection in this manuscript is downregulation of IFNg receptor, which is a well-established mechanism in TB and other diseases. The difference between the present study and previous work on subject is that this study suggests this defect is due to downregulation of IFNGR2 instead of IFNGR1. However, they do not provide an adequate explanation for why they do not recapitulate the reduction in IFNGR1 expression that has been observed across a number of studies examining type I interferon-mediated inhibition of IFNg signaling and do not provide a molecular mechanism for the negative regulation of IFNGR2 expression. Establishing a molecular mechanism that explains the reduction in IFNGR2 expression is critical given that impaired IFNg sensing is the major focus of this manuscript.

Thank you for this comment. As described in the response to Reviewer 3's second point below, we acknowledge that previous studies revealed the importance of IFNγ signaling in certain myeloid subsets under different conditions. Building on these studies, our study further revealed differential IFNγ responsiveness across distinct lung mononuclear subsets and identified an IFNγ-hyporesponsive subset, MNC1, which we previously identified as the Mtb-permissive subset (ref 8). Linking these observations, the monocyte-derived cells that are hyporesponsive to IFNγ harbor the most live intracellular Mtb. We also demonstrated that Type I interferon is one mechanism that regulates IFNγ responsiveness during Mtb infection. In addition, we provide a proof-of-concept demonstrating that prior immunity-mediated enhancement of IFNγ responsiveness in monocyte-derived cells can enhance Mtb control, providing a potential strategy for host-directed TB therapy and vaccine design.

Reviewer 3 is correct that Type I IFN downregulates IFNGR1, which is important for sensing IFNγ. Indeed, our flow cytometry results show that IFN beta decreases IFNGR1 surface levels in BMDM, consistent with prior studies (Response Figure 1) (ref 22-24). Interestingly, we found that IFN beta also reduces IFNGR2. However, the inhibition of IFNGR by Type I IFN is not complete, and IFNAR depletion did not fully restore IFNγ response in MNC1 (Figure 4), suggesting that, in addition to Type I IFN, additional mechanisms contribute to the impaired IFNγ responsiveness and responses in MNC1 during Mtb infection.

Mtb possesses multiple mechanisms of inhibiting responses to IFNγ. Previously, our group and others reported that Mtb can inhibit cultured macrophages' response to IFNγ through TLR2 and IL6 signaling (ref 39-47), and suppression of signaling downstream of IFNGR/STAT1 (ref 46-47)

(lines 241-246). Thus, we hypothesize that TLR2 and IL6 signaling may synergize with Type I IFN to impair IFNγ responsiveness in MNC1 cells. We have modified the Discussion section to include these points, read as "Since our group and others reported that Mtb can inhibit cultured macrophages' response to IFNγ through TLR2 and IL6 signaling, further studies are required to determine whether these mechanisms synergize with type I IFN to impair IFNγ responsiveness and responses in vivo during Mtb infection." (lines 287-290).

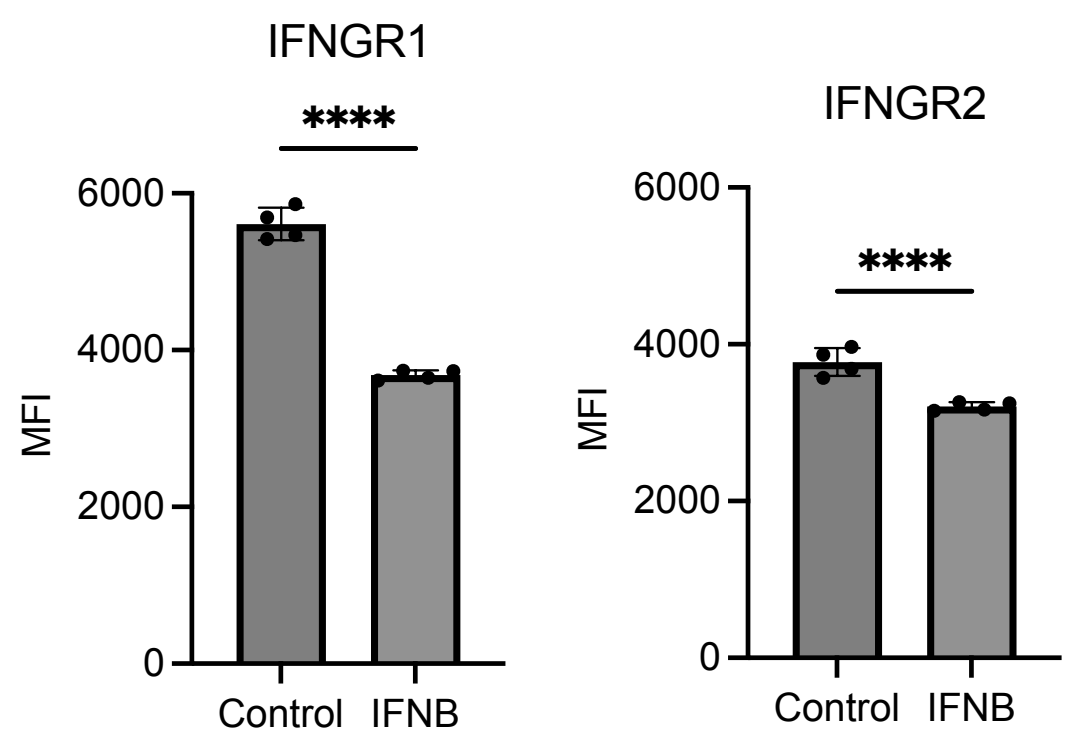

Response Figure 1: IFN beta (IFNB) reduces surface levels of IFNGR1 and IFNGR2 in BMDM. Results are presented as mean ± SD. ****p<0.0001 by unpaired t tests.

Regarding the novelty claims made by the authors, I cited multiple papers that directly assess and compare IFNg signaling across myeloid cell populations during TB infection in my initial review (e.g. PMID: 32544188, 38236787, 38029747, and 40736456). The authors address this lack of novelty by stating that "previous studies established the general importance of IFNg in these broad populations" but then make a novelty claim that they are the first to examine differences in the IFNg responsiveness across lung myeloid subsets. Perhaps the authors should re-read the manuscripts cited, as the manuscripts did look at IFNg signaling differences between lung myeloid populations thereby negating their novelty claim.

We appreciate this comment and have carefully re-read the four cited papers as suggested. We summarized the macrophage IFNγ -related findings from these four papers below.

(1) PMID- 32544188 Lee et al performed GeneChip analysis and found that at 21 days post-infection (dpi), Mtb-infected myeloid subsets (e.g., alveolar macrophages (AM)) upregulate IFNγ responses compared to uninfected counter subsets, indicating that T cell recognition of Mtb-infected cells may be driving IFNγ responses.

(2) PMID-38236787: Mai et al compared transcriptional responses in AM from control, scBCG and coMtb mice pre-aerosol challenge or at 24 hours post-infection. They revealed an enrichment of Interferon Alpha/Gamma Response pathways in AM from scBCG and coMtb mice compared to AM from control mice.

(3) PMID-38029747: Kotov et al. used scRNA-seq to reveal mouse strain-specific differences of IFNγ signaling in myeloid subsets. They show that there is a higher frequency of IFNγ -responsive

cells in interstitial macrophages (IM) of C57BL6 (SP140+/+) mice than in the same subset of Mtb-hypersusceptible SP140-/- mice at 25 dpi. They observed a similar result for AM. Please note that the expression strength of IFNγ signature genes in naive mice was used as the threshold for classifying cells as IFNγ-responsive, which is a binary separation and does not indicate relative gene expression differences across multiple subsets.

(4) PMID-40736456: Gern et al used Mtb-suceptible C3HeB/FeJ mice and performed scRNA-seq. They revealed increased outgoing *Ifng* signals from CD4 and CD8 T cells to multiple myeloid subsets (e.g. AM, monocyte-derived cells (MDC)) in the setting of CoMtb, suggesting enhanced activation of myeloid cells by T cells.

Therefore, these studies revealed the importance of IFNγ signaling in different myeloid subsets during Mtb infection. Our work extends these findings by comparing IFNγ responsiveness (the capacity to respond to IFNγ) across lung mononuclear subsets in Mtb-infected mice at 28 dpi, a time point when T cell responses are well developed (PMID: 18158321, 38110410). Our transcriptomic analysis and functional assays identified a functional "IFNγ responsiveness bottleneck" specifically within the MNC1 subset and together with our prior report, links this finding to increases in viable Mtb in that cell subset. Our study further revealed that prior immunity can reprogram monocyte-derived cells to improve IFNγ responsiveness and that IFNγR signaling is required for CoMtb-mediated protection.

To better acknowledge that previous studies have shown the importance of IFNγ signaling, we have added additional text to the Introduction section (see lines 257-261).

In response to my initial review stating pointing out their data argues that MNC2 actually have more IFNg signaling than AMs, the authors explained that they rank IFNg responsiveness as AM > MNC2 > MNC1. There arguments did not help clarify the matter as they then state “MNC2 cells express higher mRNA levels of IFNg pathway genes…compared to AM” in the next sentence and even suggest that “MNC2 may be exposed to more IFNg than AM cells” later in the paragraph. Potentially, their hierarchy of IFNg signaling does not match their own mRNA expression data because of “some key genes” being higher in AM than MNC2 at the protein level, but they did not clarify which key genes. This is particularly confusing as they show in supplementary figure 5 that MHC II protein, a key IFNg regulated gene, is consistently higher in MNC2 cells than AMs at both evaluated time points of in vivo Mtb infection. Thus, their own data demonstrate that MNC2 cells undergo more IFNg signaling than AMs during in vivo Mtb infection. This key point undermines the argument that MNC poorly control TB infection relative to AMs due to less IFNg signaling, a major focus of the paper. The authors need to provide additional in vivo evidence for greater IFNg signaling in AMs compared to MNC2 cells if they want to make this argument. This is especially important as the majority of their data supporting their IFNg signaling hierarchy depends on ex vivo stims with IFNg. These ex vivo experiments measure what would happen if the cells sense IFNg, but does not measure whether the cells actual do sense IFNg in the lungs of infected mice, and therefore cannot establish the hierarchy of IFNg signaling in vivo during TB infection.

Thank you for this insightful comment. We appreciate the opportunity to clarify the relationship between our observed hierarchy of IFNγ responsiveness and the in vivo expression of MHC-II.

We distinguish between IFNγ responsiveness (the intrinsic capacity of a cell to sense and signal) and the IFNγ response (the cumulative outcome of that signaling). Our hierarchy of AM > MNC2 > MNC1 refers specifically to the cells' intrinsic responsiveness, as measured by our ex vivo stimulation assays where cytokine concentrations are equalized. The four cited papers mentioned by the reviewer above, and this study, have shown that all these myeloid subsets can sense IFNγ in the lungs of Mtb-infected mice. We further show that AM and MNC2 cells can sense and respond to IFNγ better than MNC1 cells, the most Mtb-permissive subset.

As the reviewer noted, the hierarchy of signaling protein levels does not always mirror mRNA levels in AM and MNC2. Protein expression can be regulated by post-transcriptional and post-translational mechanisms. For example, we previously reported that neutrophils express higher levels of lysosome genes than MNC1 cells but possess lower levels of lysosome proteins and functional activity (PMID: 38701094). Similarly, the post-translational modification of IFNγ receptors (IFNGR) and downstream mediators like STAT1 and IRF1 are critical in determining the actual signaling output (PMIDs: 29921905, 30318148).

The observation that MNC2 cells express more MHC II than AM at later time points (42 and 56 dpi), despite AM having higher intrinsic responsiveness, likely reflects differences in the lung microenvironment. While AM are localized primarily in the alveolar space, MNC2 cells in the lung parenchyma may be exposed to higher or more sustained concentrations of T-cell-derived IFNγ. This increased exposure could compensate for their "intermediate" intrinsic responsiveness, leading to the higher MHC II levels observed at later time points. Furthermore, while IFNγ is a major regulator of MHC II in macrophages acting through specific CIITA promoter regions (PMID 9858567, 9551976, 9184229), other cytokines such as IL-4 can also upregulate MHC II expression (Ref 44). Whether MNC2 cells sense more IL-4 or experience synergy between IL-4 and IFNγ remains an open question for future study.

The central focus of our study is the difference in IFNγ responsiveness and responses between Mtb-permissive (MNC1) and Mtb-restrictive (AM/MNC2) subsets, as we previously reported that MNC1 cells harbor a larger number of live Mtb per infected cell than AM and MNC2 cells (PMID: 38701094). Regardless of the relative hierarchy between AMs and MNC2s, our data consistently show that MNC1 cells are the most impaired by both metrics: they exhibit the lowest intrinsic responsiveness and the lowest in vivo response (MHC II), correlating with their failure to control Mtb infection.

We have added more comments in the Discussion section to include these points (lines 271-284).

REVIEWERS' COMMENTS

Reviewer #2 (Remarks to the Author):

The authors have addressed my specific comment about including reference to the culture of the bone marrow cells in the Methods.

Author response: We are pleased that our revisions have addressed all reviewer concerns. We thank the reviewers again for their time in reviewing our revised manuscript.
